# SOAT1 Activates NLRP3 Inflammasome to Promote Cancer‐Related Lymphangiogenesis and Metastasis via IL‐1β/IL‐1R‐1 Axis in Oral Squamous Cell Carcinoma

**DOI:** 10.1002/mc.23907

**Published:** 2025-03-26

**Authors:** Chengzhi Zhao, Yuhao Wang, Zhishen Jiang, Shengzhao Guo, Liru Hu, Jian Pan, Fan Dan

**Affiliations:** ^1^ State Key Laboratory of Oral Diseases & National Center for Stomatology & National Clinical Research Center for Oral Diseases & Department of Oral Surgery, West China Hospital of Stomatology Sichuan University Chengdu China; ^2^ Department of Anesthesiology, Sichuan Provincial People's Hospital University of Electronic Science and Technology of China Chengdu China

**Keywords:** cancer metastasis, lymphangiogenesis, NLRP3 inflammasome, oral squamous cell carcinoma, sterol O‐acyltransferase 1

## Abstract

Oral squamous cell carcinoma (OSCC) is a prevalent type of cancer in the head and neck region, significantly impacting patient survival rates and quality of life. Lymph node (LN) metastasis is a lead contributor to the poor prognosis associated with OSCC. SOAT1 plays a critical role in cholesterol metabolism and has been implicated in various cancers, although its specific mechanisms in OSCC are poorly understood. Additionally, NLRP3 inflammasome has been identified as a factor that promotes cancer progression by influencing various processes involved in tumor development, with its activation linked to cancer metastasis. Lymphangiogenesis enhancing cancer metastasis has been identified in OSCC, while the molecule networks of regulating it remains unclear. In our study, we found that SOAT1 is overexpressed in OSCC and promotes proliferation, migration, and invasion of OSCC cells. Knockdown SOAT1 expression impaired OSCC progression both in vitro and in vivo, and reduced the rate of LN metastasis. RNA sequencing analysis revealed that NLRP3 is a downstream regulated by SOAT1, with NLRP3 inflammasome reactivation having recovered cancer malignancy inhibited by SOAT1 knockdown. Furthermore, we revealed that IL‐1β, released by NLRP3 inflammasome activation, could directly bind to IL‐1R‐1 in lymphatic endothelial cells (LECs), and enhance tube formation capacity of LECs, indicating the potential role of NLRP3 inflammasome in promoting lymphangiogenesis and metastasis in OSCC. In conclusion, SOAT1 could promote OSCC malignancy and regulate the activation of NLRP3 inflammasome to increase the rate of lymphangiogenesis and cancer metastasis via IL‐1β/IL‐1R‐1 axis in OSCC.

## 1 Introduction

Oral squamous cell carcinoma (OSCC), with approximately 389,485 new cases and 188,230 mortalities in 2022 [1]. As a prevalent form of squamous cell carcinoma in the region of head and neck, OSCC seriously affects the survival rate and quality of life of patients [2]. Lymph node (LN) metastasis, as the main pathway for OSCC metastasis, is closely related to the poor prognosis of patients [3, 4]. Furthermore, LN metastasis is also closely linked to the recurrence of OSCC [5, 6]. Therefore, investigating the mechanisms underlying LN metastasis in OSCC is crucial for improving patient survival rates. However, the specific mechanisms of LN metastasis of OSCC remain unclear.

Sterol O‐acyltransferase 1 (SOAT1), also known as acetyl‐CoA acetyltransferase 1 (ACAT1), plays an important role in maintaining cellular cholesterol homeostasis by catalyzing the conversion of cholesterol and long‐chain fatty acids into cholesterol esters in cells [7]. Recent research has highlighted the involvement of SOAT1 in cancers [7, 8]. In gastric cancer, SOAT1 was highly expressed in cancerous tissues, associating with advanced tumor stage, lymph node metastasis, and poor prognosis [9]. A proteomics analysis found that high expression of SOAT1 was a signature specific to the S‐III subtype, which alters the distribution of cellular cholesterol, and suppresses the proliferation and migration of hepatocellular carcinoma effectively [10]. In pancreatic ductal adenocarcinoma, SOAT1 is considered as a key player to promote PDAC in sustaining the mevalonate pathway by converting cholesterol to inert cholesterol esters [11]. However, the role of SOAT1 in OSCC has rarely been explored. Given the heterogeneity of tumors, to study the expressions and potential mechanisms of SOAT1 in OSCC may be of key significance for the treatment of OSCC.

NLR family pyrin domain containing 3 (NLRP3) inflammasome is a multimeric cytosolic protein complex that assembles in response to cellular perturbations. The activation finally leads to the maturation and release of IL‐1β and IL‐18 [12]. In a pan‐cancer analysis, 15 out of 24 cancers exhibited significantly different expression of NLRP3‐inflammasome‐related genes between normal and tumor samples [13]. Moreover, as the mediator of lymphocytes and macrophages as well as a predictive biomarker of cancer immunotherapy response, NLRP3 inflammasome has shown its prognostic and therapeutic potentials [13, 14]. Besides, NLRP3 has been implicated in promoting tumor metastasis [15, 16, 17], indicating the critical role of NLRP3 inflammasome in tumor progression.

As the initial stage of LN metastasis, cancer‐related lymphangiogenesis facilitates the dissemination of tumor cells to lymphatic vessels, ultimately causing tumor distant colonization and metastasis [18, 19]. In OSCC, lymphangiogenesis was found closely related to tumor LN metastasis. Some studies have begun to uncover the molecular mechanisms regulating lymphangiogenesis in OSCC, and have demonstrated the links between downregulating tumor lymphangiogenesis and inhibiting tumor growth [20, 21, 22, 23]. While the mechanisms of lymphangiogenesis in OSCC still remain unclear. Cytokines secreted from cancer cells and immune cells play vital roles in tumor microenvironment (TME), regulating multiple processes including tumor growth, lymphangiogenesis, and metastasis [24, 25]. A study on breast cancer found that continuous inhibition of IL‐1 activity could inhibit breast tumor growth and progression to bone metastasis in vivo [26]. Higher levels of many cytokines have also been detected in PDAC patients than in reference individuals or pancreatitis patients, and their roles related to metastasis was also confirmed [27]. Tumor‐associated macrophages (TAMs) could secrete IL‐1β or Lipocalin‐2 to promote lymphangiogenesis. Generally, these cytokines create a proinflammation TME, which enhances cancer progression, including promoting lymphangiogenesis [25]. While this process in OSCC remains unclear.

In this study, we found the higher expression of SOAT1 in OSCC tissues and confirmed that high expression of SOAT1 is associated with patients' poor prognosis and OSCC malignancy. SOAT1 knockdown significantly inhibited the aggressive behavior of OSCC cells. In addition, SOAT1 knockdown also inhibited tumor growth and metastasis in vivo. Through RNA sequencing, we confirmed NLRP3 was positively regulated by SOAT1, and confirmed the importance of NLRP3 inflammasome activation in OSCC. By releasing IL‐1β, OSCC cells enhance tube formation capacity of LECs via IL‐1β directly binding to IL‐1R‐1 in LECs, suggesting the potentials of IL‐1β/IL‐1R‐1 axis in promoting cancer‐related lymphangiogenesis and LN metastasis of OSCC.

## 2 Materials and Methods

### 2.1 Clinical Samples and Data Collection

51 paraffin‐embedded, pathologically confirmed OSCC tumor samples and 19 paired fresh frozen cancer and matched para‐cancer tissues were obtained from the Department of Pathology, West China Hospital of Stomatology, Chengdu, China. The ethics committee of West China Hospital of Stomatology has approved the testing of human samples for this study (Ethics Approval Number: WCHSIRB‐D‐2023‐198) and all patients have been provided written informed consent.

### 2.2 Data Collection and Website Analysis

The expression of SOAT1 in HNSCC and normal tissues from TCGA database were analyzed on TIMER2.0 (https://timer.cistrome.org/). Based on the expression of SOAT1 in TCGA database, survival analysis was performed, and Kaplan‐Meier curve was drawn via GEPIA (http://gepia.cancer-pku.cn/). To analyze the correlation of SOAT1 and NLRP3 with lymphangiogenesis, classic lymphangiogenesis‐related genes LYVE‐1, VEGFC, and VEGFR3 were selected, and whole gene correlation analysis procedure was performed via TIMER2.0‐Exploration‐Gene_Corr Modules.

### 2.3 Cell Culture

The cell lines were from the State Key Laboratory of Oral Diseases, West China Hospital of Stomatology. Among them, Human lymphatic endothelial cells (HLECs) were cultured in ECM (supplemented with 10% FBS, 1% ECGS, 1% penicillin/streptomycin(P/S), ScienCell, USA). Other cells were cultured in DMEM (supplemented with 10% FBS, 1% P/S, Gibco, USA). All cells were cultured in a humidified incubator with 5% CO_2_ at 37°C.

### 2.4 Silencing RNA (siRNA) Interference

To knockdown the expression of SOAT1 in OSCC cells, SOAT1 siRNA sequence was designed and generated by Tsingke Biotech Company (Shanghai, China). Lipofectamine 3000 Transfection Reagent (Thermo Fisher, USA) was used for transfection of siRNAs according to the manufacturer's instructions. All siRNAs sequences in this study are provided in Table S1.

### 2.5 RNA Extraction and Real‐Time Quantitative Reverse Transcription (qRT‐PCR)

Total RNA from cells was extracted with RNA extraction kit (Foregene, China) and NanoDrop (Thermo Fisher, USA) was used for RNA concentration measuring. Synthesis of complementary DNA (cDNA) was performed using PrimeScript FAST RT reagent Kit with gDNA Eraser (Takara, Japan). qRT‐PCR analysis was conducted using 2 × SYBR Green qPCR Master Mix (Vazyme, China). Relative expression was assessed via the 2^–ΔΔCt^ method, with GAPDH as a normalization control. The primer sequences for qRT‐PCR are listed in Table S2.

### 2.6 Western Blot

Total protein in cell lysate was extracted using radioimmunoprecipitation assay (RIPA) buffer (epizyme, China) with 1% phenylmethyl sulfonyl fluoride (PMSF, epizyme, China), followed by centrifuging at 12,000 g for 15 min at 4°C. Bicinchoninic acid (BCA) assay was performed to measure and adjust concentrations of protein to 15 μg. Total proteins were separated via SDS‐PAGE electrophoresis, then transferred onto polyvinylidene difluoride (PVDF) membranes, followed by blocking with 5% skim milk for 1 h. Primary antibodies were incubated overnight at 4°C. Blots were probed with proper secondary antibodies and detected by a chemiluminescent imaging system (Bio‐Rad, USA). Primary antibodies used in western blot includes SOAT1 (1:1000, ab307597, abcam, USA), NLRP3 (1:1000, ab270449, abcam, USA), β‐actin (1:10000, EM21001, HuaBio, China). Secondary antibodies include HRP Conjugated Goat anti‐Rabbit/Mouse IgG polyclonal Antibody (1:50000, HA1001/HA1006, HuaBio, China).

### 2.7 Enzyme‐Linked Immunosorbent Assay (ELISA)

To quantify the secretion of IL‐1β in OSCC cells, ELISA was performed using a human IL‐1β ELISA kit (EH0006, Youkelife, China) according to the manufacturer's instructions. Briefly, after centrifuging to remove cell pellets and debris, the supernatant from OSCC cells was collected and were immediately stored at −80°C for following experiments. The standard samples were diluted according to the instructions. After adding the detection antibody, the samples were incubated at room temperature for 1 h at a microplate shaker set at 500 rpm. After thoroughly washing off unbound antibodies, Streptavidin‐HRP was added to each well and incubated at room temperature for an additional 30 min at a microplate shaker set at 500 rpm. Finally, TMB substrate was added, and the reaction was stopped when a significant color change was observed within standard samples. Absorbance at 450 nm was immediately measured using a microplate reader.

### 2.8 Cell Viability and Colony Formation Assay

Cell viability was measured by ki‐67 staining, experimental details was described in section Immunofluorescence (IF) staining. For colony formation assay, cells were digested and counted at 1000 per well, then seeded in 12‐well plates and cultured for 7 days. Then, the plates were fixed by 4% paraformaldehyde (PFA) and stained with 0.1% crystal violet staining solution for 20‐30 min. Colony formation images was recorded by camera.

### 2.9 Wound Healing Assay

Cells were seeded in 6‐well plates (5 × 10^4^ cell/well). A 200 µL pipette tip was used to introduce a scratch wound. After scratching and washing with PBS, cells were cultured for 24 h in serum‐free medium, wound healing images were captured at 0 and 24 h timepoint.

### 2.10 Transwell Migration and Invasion Assay

For transwell migration assay, cells were incubated in upper chambers cultured with serum‐free medium (2 × 10^4^ cell/well), lower chambers were added medium containing 10% FBS. After 24 h, upper chambers were taken out, fixed with 4% PFA, and then stained with 0.1% crystal violet staining solution for 20–30 min. For transwell invasion assay, upper chamber was precoated with 1% Matrixgel (Corning, USA), then cells were seeded in upper chamber cultured with serum‐free medium (2 × 10^4^ cell/well). Lower chambers were added medium containing 10% FBS. After culturing 24–48 h, upper chambers were taken out, fixed with 4% PFA, and then stained with 0.1% crystal violet staining solution for 20–30 min.

### 2.11 HLECS Tube Formation and Migration Assay

For HLECs tube formation assay, HLECs were seeded in 48‐well plate (2 × 10^4^ cell/well) which was precoated with Matrixgel (Corning, USA) and cultured with conditional media from cancer cells and cultured for 12 h. For evaluating HLECs' migration ability, trasnswell chamber were applied. Briefly, HLECs were suspended with serum‐free medium and then seeded in upper chamber (5 × 10^4^ cell/well). Cancer cells were seeded in lower chamber (1 × 10 ^5^ cell/well). The other details were the same with cancer cell migration assay. After coculture for 24 h, upper chambers were taken out.

### 2.12 Lentivirus Transfection

Lentiviral vector containing SOAT1‐specific siRNAs (shSOAT1) were purchased from Hanbio Company (Shanghai, China). HSC‐3 OSCC cells were seeded in 6‐well plate and used to transfect lentiviral (2 × 10^5^ cell/well). The transfection efficiency was evaluated based on GFP fluorescence density using a fluorescence microscope (Leica, Germany). Puromycin (Hanbio Biotech, Shanghai, China) were used for cell selection after 48–72 h post transfection according to manufacturer's protocol. SOAT1 knockdown efficiency was evaluated by qRT‐PCR and Western blot. Sequences of siRNA used for shRNA were recorded in Table S1.

### 2.13 RNA Sequencing and Bioinformatics Analysis

Lentivirus transfected, and knockdown efficiency confirmed Cal‐27 cells as well as Cal‐27 cells in control were seeded in 6‐well plate (5 × 10^4^/well). RNA was extracted from Cal‐27 cells using TRIzol Reagent (Thermo Fisher, USA). Extracted RNA were frozen in liquid nitrogen and stored at −80°C. The mRNA in the lysate was extracted and its integrity and total amount were measured (Agilent, USA). The cDNA libraries were then prepared and sequenced (Illumina, USA) by Novogene (China).

Sequencing raw data were firstly filtered by removing reads containing adapter, reads containing ploy‐N and low‐quality reads to get clean data for further analysis. Index of the reference genome was built using Hisat2 v2.0.5 and paired‐end clean reads were aligned to the reference genome using Hisat2 v2.0.5. FeatureCounts v1.5.0‐p3 was used to count the reads numbers mapped to each gene. And then FPKM of each gene was calculated based on the length of the gene and reads count mapped to this gene.

For differentially expressed genes (DEGs) between shNC‐ and shSOAT1‐ Cal‐27 cells, DEseq. 2 R package (1.20.0) were used. Genes with a *p* < 0.05 and absolute foldchange of 1 were set as the threshold for significantly differential expression. Gene Ontology (GO) enrichment analysis and Kyoto Encyclopedia of Genes and Genomes (KEGG) enrichment analysis of DEGs was then performed by the clusterProfiler R package. Gene Set Enrichment Analysis (GSEA) were also performed to show the significant consistent difference between two groups. Predefined gene set were downloaded from GSEA database (https://www.gsea-msigdb.org/gsea/msigdb/index.jsp).

### 2.14 Immunofluorescence (IF) Staining

Cells were seeded in confocal disk (5 × 10^4^cell/well). After culturing 24 h, cells were fixed with 4% PFA and permeabilized with 0.5% Triton X‐100. After 1 h incubation with 1% BSA at room temperature, primary antibodies were incubated at 4°C overnight. Then proper second antibodies were used as follows. Nuclei were stained with DAPI. Images were obtained by confocal microscopy (FV3000, Olympus, Japan). Primary antibodies used in this section include anti‐Ki67 (1:500, HA721115, Huabio, China) and anti‐NLRP3 (1:200, ab270449, abcam, USA). FITC‐ or iFluor 594‐conjugated secondary antibodies were selected (1:500, HA1124/HA1122, Huabio, China).

### 2.15 Hematoxylin & Eosin (H&E) and Immunohistochemistry (IHC) Staining

The paraffin sections of patients and animal tongue tissues were first dewaxed using a gradient of ethanol. For H&E staining, the cell nuclei were stained with hematoxylin, followed by eosin staining. For IHC staining, after dewaxing, the slide was first subjected to antigen retrieval using EDTA antigen retrieval solution (Beyotime, China). After retrieval, experiments were conducted according to the instructions of the immunohistochemistry kit (ZSGB‐bio, China). 3,3’‐diaminobenzidine (DAB, ZSGB‐bio, China) was used for staining, and hematoxylin was used for cell nuclei staining. Finally, the sections were sealed with neutral resin (Solarbio, China). mIHC staining was conducted according to manufacturer's protocol, and slided was sealed with antifade mounting medium with DAPI (Beyotime, China). Primary antibodies include: anti‐SOAT1 (1:400, ER1916‐93, Huabio, China), anti‐NLRP3 (1:200, ET1610‐93, Huabio, China), anti‐IL‐1β (1:400, ab283818, abcam, USA), anti‐LYVE‐1 (1:600, ab218535, abcam, USA).

### 2.16 Immunoreactivity Score (IRS) Grading

The criteria of IRS grading were described preciously [28]. In brief, positive cell rate and staining intensity was recorded. 0‐5% positive cell rate ranks 0 point, 6%–25% ranks 1 point, 26%–50% ranks 2 point, 51%–75% ranks 3 point, 76–100 ranks 4 point. No staining ranks 0 point, light staining ranks 1 point, moderate staining ranks 2 point, dark staining ranks 3 point. The multiple result of positive rate score and staining intensity score is the final IRS score.

### 2.17 Tongue Orthotopic Xenograft Model Establishment and Fluorescence Imaging

Before experiment, the protocols of animal experiments and animal care approaches has been reviewed and approved by the ethics committee of West China Hospital of Stomatology (Ethics approval number: WCHSIRB‐D‐2023‐324). For animal experiment, 10 4‐week‐old female BALB/c nude mice were randomized into two groups and were raised in specific pathogen‐free (SPF)‐breeding rooms. Mice were settled for 1‐week adaptive feeding before formal experiments. For model establishment, the mice were anesthetized with isoflurane (RWD Life Science, China), lateral margin of tongue was then injected with GFP‐shNC‐ or GFP‐shSOAT1‐ transfected HSC‐3 cells (1 × 10^7^ cell/mouse) suspended in 30 μL media. The body weight of mice was measured every 5 days. For tumor growth estimation, as the blocking of mandible jaw, tongue tissues were hard to detect directly in vivo, thus we didn't conduct tumor growth assay until mice were euthanized at Day 20 after the injection, and their tongue tissues were harvested and detected immediately for GFP fluorescence via in vivo imaging system (AniView100, BLT Biotech., China). To estimate LV metastasis, in vivo imaging system (AniView100, BLT Biotech., China) was used before mice were euthanized since neck regions of mice were not blocked as we described that to tongues. Tongue tumors and cervical LNs were collected for following experiments. LNs volume (mm^3^) was identified as width (mm)^2^ × length (mm)/2.

### 2.18 Statistics Analysis

Means comparisons were performed using Student's *t*‐test for two groups or one‐way ANOVA for more than two groups. GraphPad Prism 8.0 (GraphPad Software) was used for processing all data and values were presented as means ± SD. *p < 0.05 was the significance threshold. Correlation analysis for clinical parameters of patients were analyzed using the Pearson *χ* ^2^ test. The survival rates were assessed using the Kaplan‐Meier method, and statistical significance between groups was estimated by log‐rank test. All experiments were repeated at least for three times.

## 3 Results

### 3.1 SOAT1 Is Upregulated and Correlated With LN Metastasis in OSCC Patients

**Figure 1:**
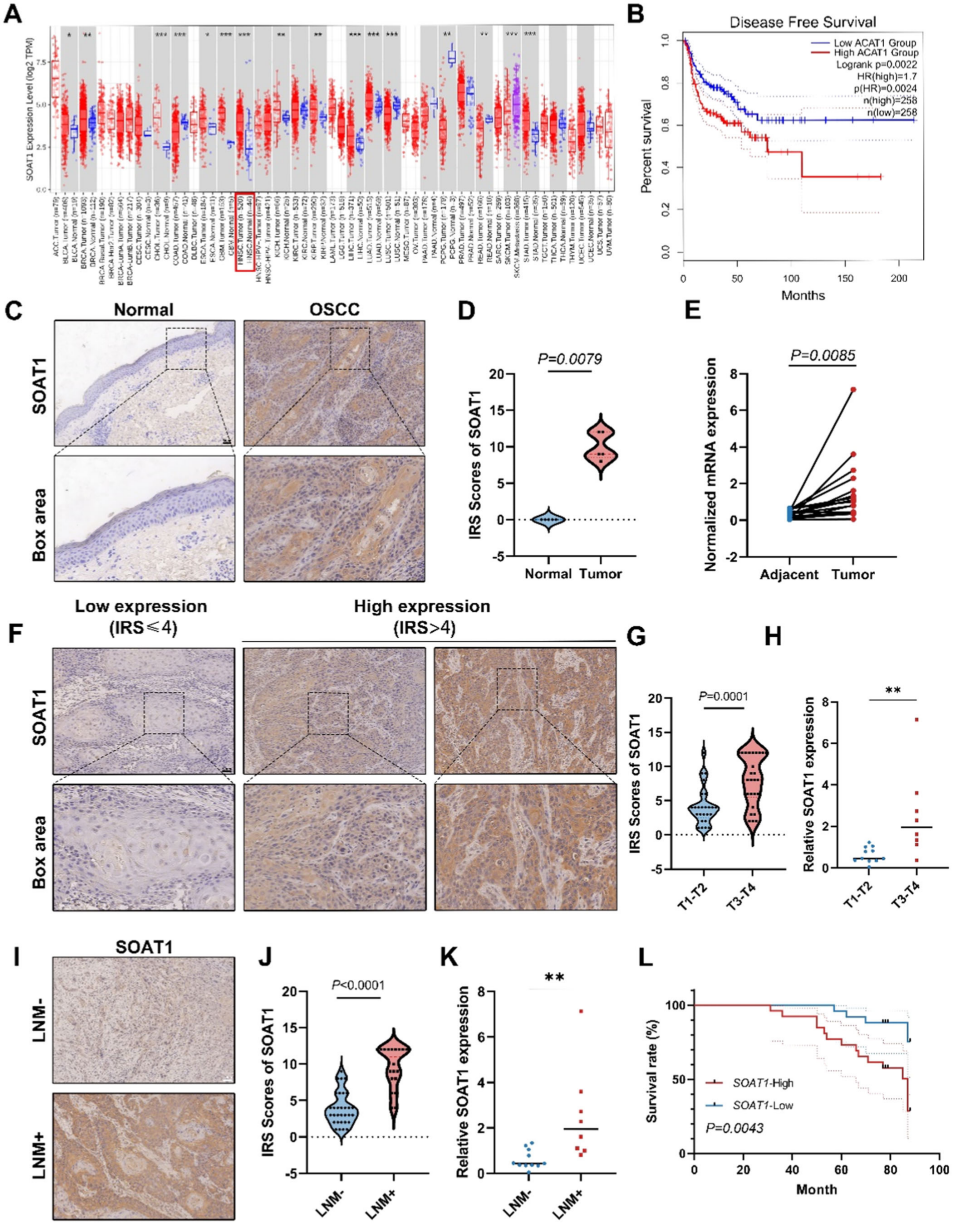
SOAT1 is highly expressed in OSCC and associated with LN metastasis and poor prognosis. (A) Pan‐cancer analysis of SOAT1 expression according to TIMER2.0 database. The expression in HNSCC was highlighted in red rectangle. (B) Disease‐free survival curve revealed the correlation of the survival and SOAT1 expression in OSCC patients according to TIMER2.0 database. (C and D) Representative IHC images showed the SOAT1 expression in tumor regions and normal epithelium. (E) Paired *t*‐test revealed the SOAT1 mRNA levels in OSCC tissues and adjacent noncancerous tissues (*n* = 19). (F and G) Representative IHC images of SOAT1 expression based on IRS scoring and its correlation to clinical stages of OSCC patients. (H) The correlation of SOAT1 mRNA expression and clinical stages of OSCC patients. (I and J) Representative IHC images of SOAT1 expression and its correlation to LN metastasis of OSCC. (K) The correlation of SOAT1 mRNA expression and LN metastasis of OSCC patients. (L) The correlation of the survival rate and SOAT1 expression within the IHC cohort by Kaplan–Meier survival analysis. **p < 0.01. Scale bar of IHC figure = 50 μm.

To investigate the role of SOAT1 in OSCC, we first analyzed data from the TCGA database to validate the expressions of SOAT1 in OSCC patients. The results indicated that SOAT1 expression levels in OSCC tumor tissues (*n* = 520) were significantly higher than those in the control group (n = 44, *p* < 0.001, Figure 1A). Additionally, website survival analysis also revealed that patients with higher SOAT1 expression had significantly poorer survival outcomes compared to those with lower SOAT1 expression (*p* = 0.0024, Figure 1B). To further verify the database results, we performed IHC staining on paraffin‐embedded samples from OSCC patients who were in hospital. The results found an increased SOAT1 expression in tumor tissues compared to that in normal epithelial tissues (*p* = 0.0079, Figure 1C,D). Furthermore, we assessed SOAT1 expression levels in fresh tumor samples and matched adjacent noncancerous samples (*n* = 19) by qRT‐PCR. A significantly higher SOAT1 mRNA levels in tumor tissues compared to adjacent noncancerous tissues were detected (*p* = 0.0085, Figure 1E). To further evaluate whether SOAT1 expression is associated with clinical and pathological characteristics of tumors, IHC staining on paraffin specimens from OSCC patients and an immunoreactive score (IRS) assessment was performed. An IRS score of ≤ 4 was classified as low SOAT1 expression, while an IRS score of > 4 indicated high expression (Figure 1F). The results showed that all included OSCC patients (*n* = 51) displayed positive SOAT1 expression in their tumor tissues. When analyzing the correlation between SOAT1 expression levels and clinical‐pathological parameters, we found a statistically positive correlation with clinical stage (*p* < 0.0001) and lymph node metastasis (*p* < 0.0001). However, no significant differences were observed with age (*p* = 0.121), sex (*p* = 0.208), or pathological grading (*p* = 0.078) (Table 1, Figure 1G,J). qRT‐PCR analysis of patient SOAT1 mRNA levels demonstrated a similar trend, with higher expression levels in patients at clinical stages T3‐T4 and those with lymph node metastasis (Figure 1H,K). Moreover, patients with high SOAT1 expression exhibited worse clinical prognosis in our cohort (Figure 1L). All in all, SOAT1 were highly expressed in OSCC patients and indicated a potentially worse prognosis for patients.

**Table 1:** Association between the expressions of SOAT1 and clinical characteristics of OSCC patients.

Clinical parameters	Cases (*n*)	SOAT1		χ^2^	*p* value
		Low expression (IRS ≤ 4)	High expression (IRS＞4)		
Age (years)					
＜ 60	24	9	15	2.407	0.121
≥ 60	27	16	11
Sex					
Male	31	13	18	1.587	0.208
Female	20	12	8
Clinical stage					
T1–T2	25	19	6	14.284	**＜ 0.0001**
T3–T4	26	6	20
Pathology grade					
Grade I	32	19	13	5.107	0.078
Grade II	16	6	10
Grade III	3	0	3
Lymph node metastasis					
Yes	20	2	18	20.046	**＜ 0.0001**
No	31	23	8

*Note:* Bold values are statistically significant.

### 3.2 SOAT1 Knockdown Impairs Proliferation, Migration, and Invasion of OSCC Cells

**Figure 2:**
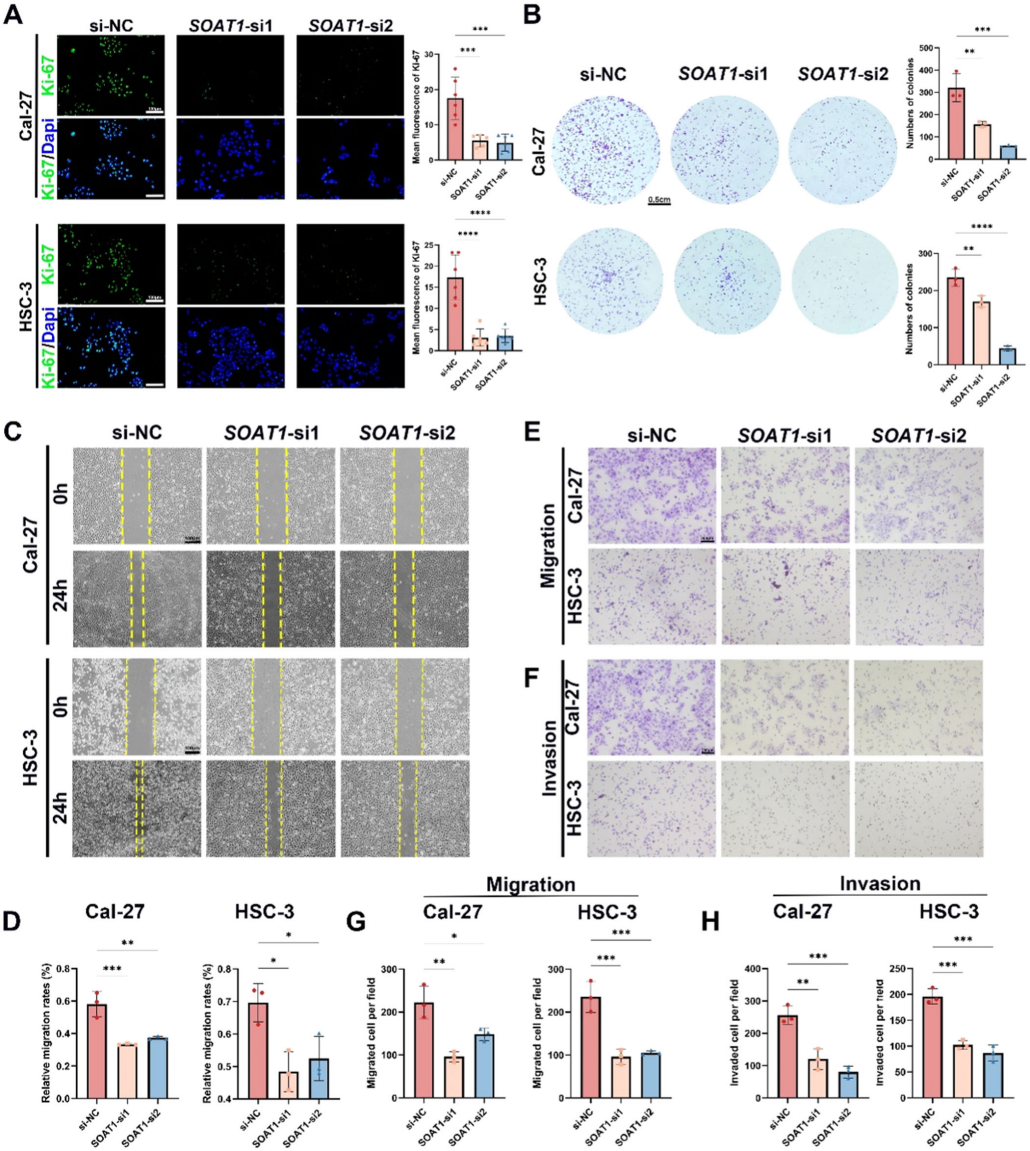
SOAT1 promotes cell proliferation, migration and invasion in OSCC cancer cells. (A) Representative images of Ki‐67 staining showed the impact of SOAT1 on the proliferation of Cal‐27 and HSC‐3 OSCC cells. Scale bar = 100 μm. (B) The impact of SOAT1 on the colony formation of Cal‐27 and HSC‐3 OSCC cells. Scale bar = 0.5 cm. (C and D) The impact of SOAT1 on migration of Cal‐27 and HSC‐3 OSCC cells by wound healing assay. Scale bar = 100 μm. (E and G) Representative images of transwell migration assay showed the impact of SOAT1 on the migration of Cal‐27 and HSC‐3 OSCC cells. Scale bar = 200 μm. (F and H) Representative images of transwell invasion assay showed the impact of SOAT1 on the invasion of Cal‐27 and HSC‐3 OSCC cells. Scale bar = 100 μm. *p < 0.05, **p < 0.01, ***p < 0.001, ****p < 0.0001.

To investigate the role of SOAT1 in OSCC, we designed siRNAs to knockdown the expression of SOAT1 in OSCC cells and confirmed knockdown efficiency of the siRNA sequences (Figure S1). The proliferation capacity of Cal‐27 and HSC‐3 OSCC cells was markedly inhibited by downregulating SOAT1 (Figure 2A). Furthermore, we assessed the colony formation ability of OSCC cells, and the knockdown of SOAT1 also significantly suppressed the colony formation potentials (Figure 2B). Additionally, the impact of SOAT1 on the migration of OSCC cells was also examined. Scratch wound assay indicated that the downregulation of SOAT1 affected the migration of Cal‐27 and HSC‐3 cells (Figure 2C,D). Moreover, we conducted migration and invasion assays using transwell chambers to reinforce the impact of SOAT1 on tumor invasiveness in Cal‐27 and HSC‐3 cells. The results showed consistency with previous results, which demonstrated the knockdown of SOAT1 significantly diminished the migration and invasion abilities of Cal‐27 and HSC‐3 cells (Figure 2E–H).

### 3.3 NLRP3 Is Downregulated in SOAT1‐Knockdown OSCC Cells

**Figure 3:**
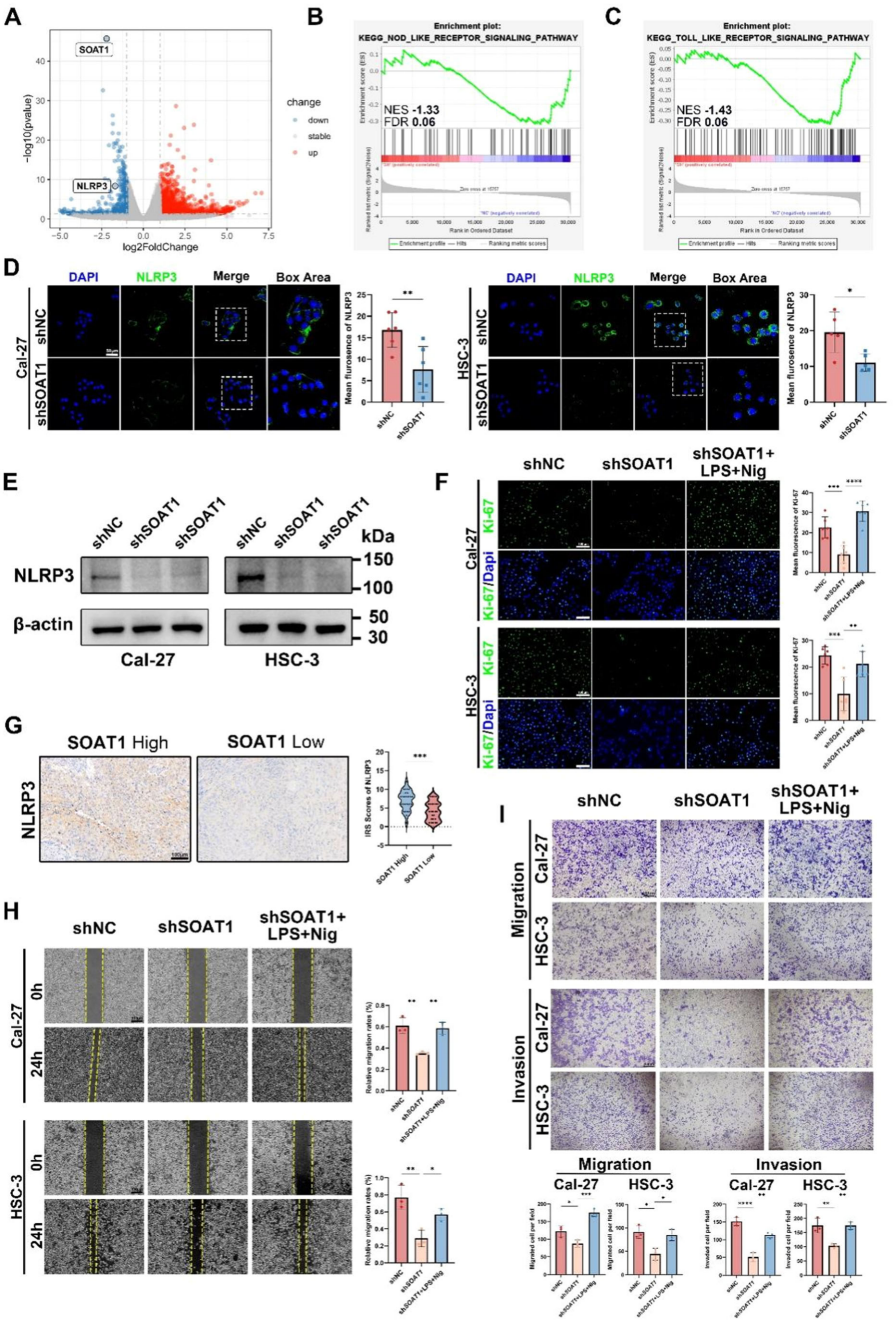
RNA‐seq reveals that NLRP3 is a downstream of SOAT1 and regulates OSCC malignancy. (A) Volcano Plot of differentially expressed genes of Cal‐27 cells after SOAT1 knockdown compared to control group. (B and C) The plots of GSEA analysis showed the enrichment score of SOAT1‐regulated genes in Toll‐like and Nod‐ like receptor signaling pathway. (D) Representative images of NLRP3 staining in shNC‐ and shSOAT1‐ OSCC cells. Scale bar = 50 μm. (E) Western blot of NLRP3 expression in shNC‐ and shSOAT1‐ OSCC cells. (F) Representative images of Ki‐67 staining showed that the impact of reactivation of NLRP3 inflammasome on the proliferation of Cal‐27 and HSC‐3 OSCC cells. Scale bar = 100 μm. (G) Representative IHC images showed the association of NLRP3 expression and SOAT1 expression in OSCC patients. Scale bar = 100 μm. (H and I) Representative images of wound healing assay and transwell migration and invasion assay showed that the impact of reactivation of NLRP3 inflammasome on the migration and invasion of Cal‐27 and HSC‐3 OSCC cells. Scale bar in wound healing assay = 100 μm. Scale bar in transwell assay = 200 μm. *p < 0.05, **p < 0.01, ***p < 0.001, ****p < 0.0001.

Aforementioned experiments have proven that SOAT1 can influence various malignant biological behaviors of OSCC cells, indicating its significant role in the development of OSCC. To further elucidate the specific mechanisms regulated by SOAT1, we performed RNA sequencing to explore the gene alterations following SOAT1 knockdown. Initially, we designed and constructed a lentivirus carrying shSOAT1, which was then transfected into Cal‐27 cells. The transfection efficacy and knockdown efficacy were both validated (Figure S2). After SOAT1 knockdown, Cal‐27 cells exhibited notable differences in gene expression (Figure S3A,B). Compared to the control group, knockdown of SOAT1 resulted in the upregulation of 235 genes and the downregulation of 734 genes in Cal‐27 cells (Figure 3A). The knockdown of SOAT1 in Cal‐27 cells significantly impacted the gene enrichment of several lipid metabolism pathways (Figure S3C,D), indicating substantial alterations in lipid metabolism within tumor cells following SOAT1 knockdown.

Furthermore, NLRP3 was found to be significantly downregulated in Cal‐27 cells following SOAT1 knockdown (Figure 3A). Since the expression of NLRP3 was closely related to Nod‐like receptor pathway and Toll‐like receptor pathway [29, 30]. Gene set enrichment analysis (GSEA) was then performed to analyze the results. And a significant reduction in genes associated with the Nod‐like receptor pathway and the Toll‐like receptor pathway after SOAT1 knockdown was noticed (Figure 3B,C and S3E,F). To validate the sequencing results, we assessed the expressions of NLRP3 in both Cal‐27 and HSC‐3 cells. The results indicated a significant decrease in NLRP3 expression levels in both shSOAT1‐ Cal‐27 and HSC‐3 cells (Figure 3D,E). Additionally, Clinical samples revealed that patients with high SOAT1 expression exhibited elevated NLRP3 expression levels, suggesting a positive correlation between SOAT1 and NLRP3 expression (Figure 3G).

To further assess the biological impact of NLRP3 alterations on OSCC cells, we re‐upregulated NLRP3 expression by using lipopolysaccharide (LPS) and Nigericin (Nig) costimulation [31]. Upon restoration of NLRP3 expression, the proliferation capacities of both Cal‐27 and HSC‐3 cells were significantly recovered (Figure 3F). Furthermore, we evaluated the migration and invasion of Cal‐27 and HSC‐3 cells as well, which was also recovered following NLRP3 inflammasome reactivation (Figure 3H,I). These findings indicate that NLRP3 inflammasome activation could alter proliferation, migration, and invasion capabilities of OSCC cells, implying the significance of it regulating OSCC.

### 3.4 SOAT1 Promotes Tumor Growth and Cervical Lymph Node Metastasis In Vivo

**Figure 4:**
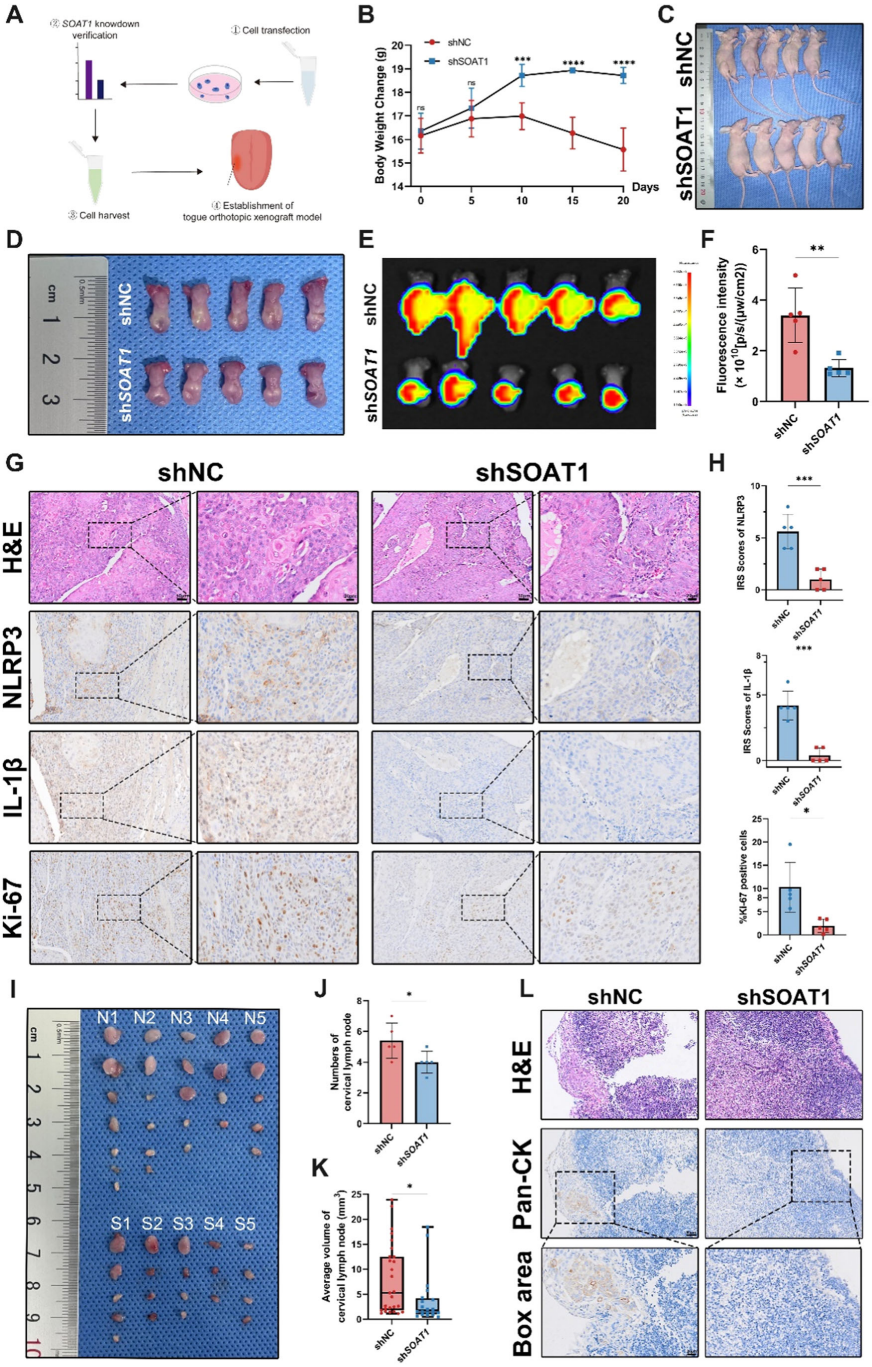
SOAT1 promotes OSCC tumor growth in vivo and is positively associated with LN metastasis. (A) Schematic diagram of tongue orthotopic xenograft model establishment in BALB/c nude mice. (B and C) Body weight changes and gross images of tumor‐implanted nude mice. (D) Dissected tongue images in shNC‐ and shSOAT1‐mice groups. (E and F) GFP fluorescence signal detection of tongues in shNC‐ and shSOAT1‐mice groups. (G and H) Representative images of H&E staining and IHC staining of NLRP3 and IL‐1β and quantitative analysis in shNC‐ and shSOAT1‐mice groups. (I) Dissected LNs in shNC‐ and shSOAT1‐ mice groups. (J and K) Quantitative analysis of LN numbers and LN volumes in shNC‐ and shSOAT1‐ mice groups. (L) Representative images of H&E and Pan‐CK IHC staining in shNC‐ and shSOAT1‐ mice groups. *p < 0.05, **p < 0.01, ***p < 0.001, ****p < 0.0001. Scale bar of IHC figure = 50 μm (20 μm for box area).

To further investigate the role of SOAT1 and NLRP3 inflammasome in the tumorigenesis and progression of OSCC, we established a tongue orthotopic xenograft model in nude mice (Figure 4A). A decline of body weight in shNC‐ mice was noticed over time compared to that in shSOAT1‐ mice (Figure 4B,C). Further dissection of the tongues showed tumor formation at the tongues' left lateral margins. While considered that the tumor may infiltrate into muscle layers, we couldn't precisely dissect tumor only. Besides, possibly due to rapid tumor growth, there were ulceration along the tongue edges exhibiting in some mice of both groups, which impeded our direct judgment of tumor formation (Figure 4D). Therefore, we detect GFP fluorescence intensity for estimating tumor size. While due to the blockage of mandible jaw, in vivo fluorescence imaging for tumor growth estimation was inaccurate, therefore we didn't measure the tumor growth until we euthanatized mice and dissected the tongues. Immediate fluorescence imaging on the dissected tongue tissue demonstrated significantly stronger GFP signals in the shNC‐ group (Figure 4E,F). This indicates that OSCC cell proliferation was more active in the shNC‐ group in vivo, leading to larger tongue tumors. To further elucidate the impact of SOAT1 on NLRP3, we embedded the tongue tissues for histological examination. H&E staining indicated the presence of tumors in the tongue tissues of both groups, with cancer cells infiltrating into tongue muscles as we predicted (Figure 4G). IHC staining for Ki‐67 revealed that OSCC cells in the shNC‐ group exhibited a higher proliferation capacity compared to those in the shSOAT1‐ group. Additionally, the expressions of NLRP3 and its downstream molecule IL‐1β were downregulated in shSOAT1‐ group (Figure 4G,H).

Next, we observed lymph node metastasis in both groups. Although mandible jaws blocked the tongue tissues, while the LNs were free from its blockage. Therefore, we initially assessed the GFP intensity in the neck regions of two groups of nude mice through in vivo fluorescence imaging to preliminarily determine the occurrence of LN metastasis. We found that the shNC‐ group displayed stronger GFP fluorescent signals in the neck region, suggesting potential LN metastasis (Figure S4). A greater number and higher volume of lymph nodes were dissected in shNC‐ group as well (Figure 4I–K). Pan‐CK positive cells were detected at the margins of the LNs in the shNC‐ group, while seldom positive cells were observed in LNs from shSOAT1‐ group, which suggests that SOAT1 knockdown may prevent OSCC from lymph node metastasis (Figure 4L).

### 3.5 SOAT1 Promotes Lymphagiogenesis via NLRP3

**Figure 5:**
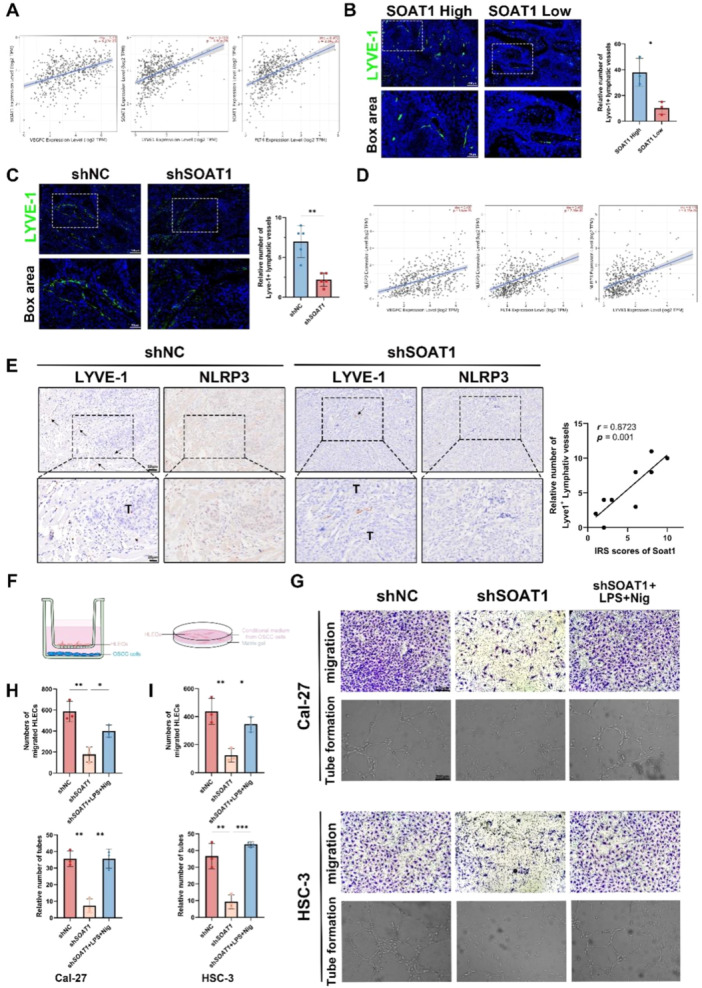
SOAT1 and NLRP3 inflammasome activation promote tube formation capacity of HLECs and cancer‐related lymphangiogenesis. (A) The correlation between SOAT1 and lymphangiogenesis‐related genes (VEGFC, LYVE‐1, and VEGFR3). (B) Representative images and quantitative analysis of IF staining of LYVE‐1 in clinical samples. Scale bar = 100 μm. (C) Representative images and quantitative analysis of IF staining of LYVE‐1 in shNC‐ and shSOAT1‐mice samples. Scale bar = 100 μm. (D) The correlation between NLRP3 and lymphangiogenesis‐related genes (VEGFC, LYVE‐1, and VEGFR3). (E) Representative images of IHC staining and correlation analysis of NLRP3 and LYVE‐1 in shNC‐ and shSOAT1‐ mice samples. Scale bar = 50 μm (20 μm for box area). T: Tumor. Arrows indicate LYVE‐1^+^ lymphatic vessels. (F) Schematic diagram illustrated cancer‐associated HLEC migration and tube formation assay. (G) Cell migration assay and tube formation assay of HLECs cultured in different groups of supernatants from OSCC cells. Scale bar = 200 μm. (H) Quantitative analysis of HLEC migration assay and tube formation assay cultured with supernatant of Cal‐27 OSCC cells. (I) Quantitative analysis of HLEC migration assay and tube formation assay cultured with supernatant of HSC‐3 OSCC cells. *p < 0.05, **p < 0.01, ***p < 0.001.

The formation of lymphatic vessels (lymphangiogenesis) is a crucial mechanism that facilitates lymph node metastasis in OSCC [20]. Therefore, we firstly aimed to investigate the potential association between SOAT1 and lymphatic vessel formation in OSCC. Initially, we conducted a database analysis to assess the relationship between SOAT1 and classic markers of lymphatic vessel formation, including LYVE‐1, VEGFC, and VEGFR3 (also known as FLT4). The results indicated a positive correlation between SOAT1 expression and lymphatic vessel formation (Figure 5A). To confirm database results, we evaluated the expression of LYVE‐1 in clinical paraffin embedded samples, revealing that patients with higher SOAT1 expression exhibited higher expression of LYVE‐1 in tumor tissues (Figure 5B). Additionally, the expression of LYVE‐1 was also found decreased in shSOAT1‐ nude mice compared to that in shNC‐ group, indicating the significance of SOAT1 in OSCC related lymphangiogenesis (Figure 5C).

To further investigate whether NLRP3 influences cancer‐related lymphangiogenesis, we analyzed the relationship between NLRP3 and lymphatic vessel formation. Database analysis indicated a positive correlation between NLRP3 and lymphatic vessel formation as well (Figure 5D). Furthermore, the positive correlation between NLRP3 and lymphangiogenesis (LYVE‐1^+^) was confirmed via IHC staining in nude mice. In shNC‐ group, the higher expression of NLPR3 was related to more density of lymphatic vessel formation and larger vessel diameter. While in shSOAT1‐ group, not only the expression of NLRP3 was decreased, but the density and length of lymphatic vessel was reduced, and a positive relationship between them was also confirmed (*r* = 0.8723, *p* = 0.001) (Figure 5E).

For validation of the role of NLRP3 in cancer‐related lymphangiogenesis, we conducted HLEC migration assay and HLEC tube formation assay in vitro. The migration and tube formation capabilities of HLEC were significantly impaired when it was cocultured with shSOAT1‐ OSCC cells. While the reactivation of NLRP3 inflammasome in OSCC cells restored the HLEC migration and tube formation capabilities, indicated the lymphangiogenesis of HLECs was dependent on the NLRP3 inflammasome activation in OSCC cells (Figure 5F–I). Taken together, these findings suggest that as the downstream of SOAT1, NLRP3 inflammasome activation positively impacted cancer‐related lymphangiogenesis in OSCC.

### 3.6 IL‐1β/IL‐1R‐1 Axis Plays Vital Functions in Regulating Tumor‐Related Lymphangiogenesis in OSCC

**Figure 6:**
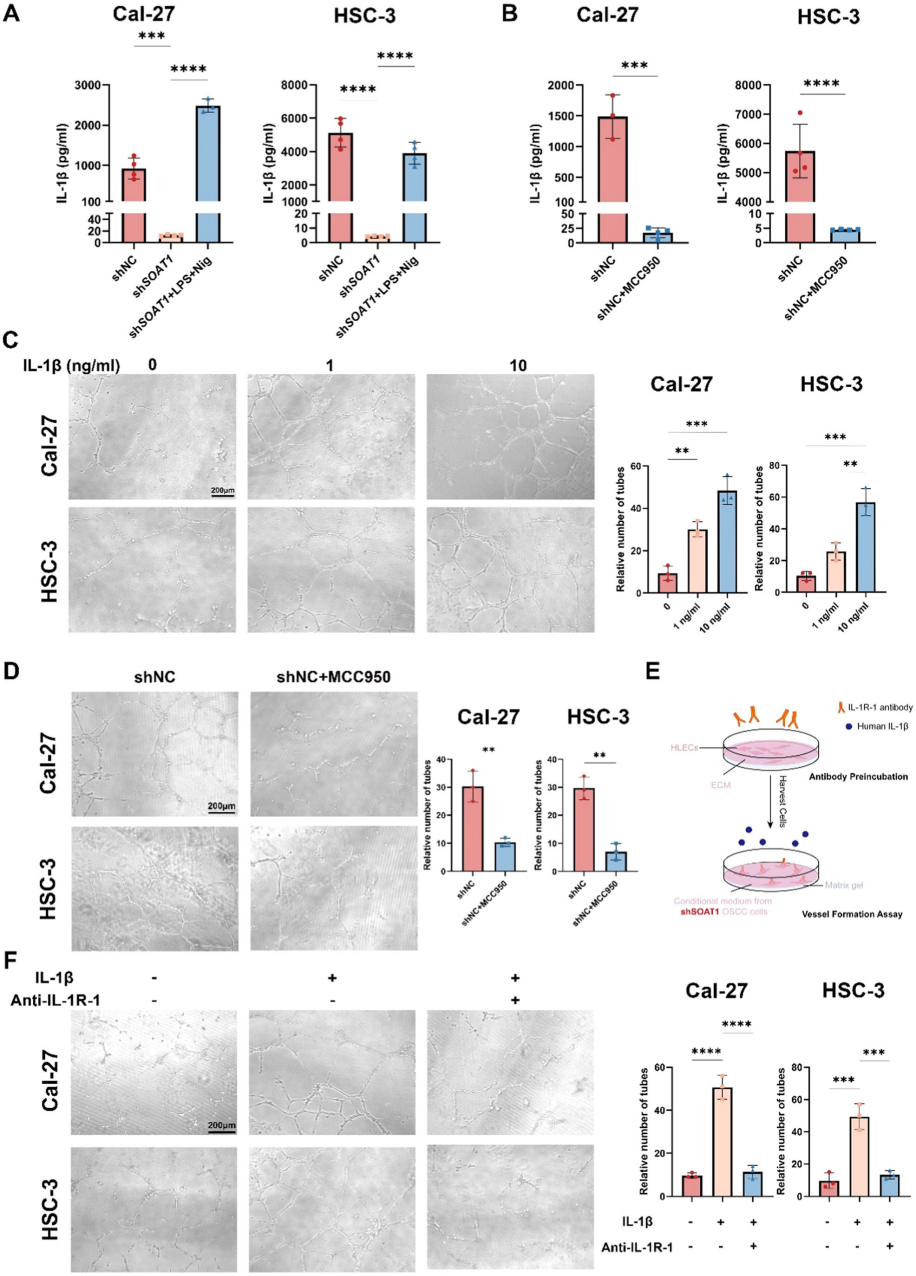
IL‐1β secreted by SOAT1‐mediated NLRP3 inflammasome activation could directly bind to IL‐1R‐1 on HLECs and promotes lymphatic tube formation. (A)IL‐1β ELISA detection in Cal‐27 and HSC‐3 OSCC cells after NLRP3 reactivation. (B) IL‐1β ELISA detection in Cal‐27 and HSC‐3 OSCC cells after the addition of MCC950. (C) Representative images of tube formation and quantitative analysis of HLECs treated with IL‐1β. (D) Representative images of tube formation and quantitative analysis of HLECs cultured with supernatant from OSCC cells treated with MCC950. (E) Schematic diagram illustrated IL‐1R‐1 neutralization and subsequent tube formation assay. (F) Representative images of tube formation and quantitative analysis of IL‐1R‐1 preneutralized HLECs cultured with shSOAT1‐ OSCC cells and treated with IL‐1β. **p < 0.01, ***p < 0.001, ****p < 0.0001. Scale bar = 200 μm.

NLRP3 is a key molecule in the NLRP3 inflammasome pathway, and inflammasome activation helps the maturation and release of IL‐1β [12]. Therefore, to determine whether OSCC cells directly alter lymphangiogenesis rather than depending on other TME cell components, we also investigated IL‐1β release from OSCC cells. We found that IL‐1β release from OSCC cells was significantly reduced when SOAT1 was knocked down. And the expression was restored when we re‐activated NLRP3 inflammasome in OSCC cells with LPS and Nig (Figure 6A). To further verify that IL‐1β release in OSCC cells was mainly attributed by NLRP3 inflammasome activation, we used MCC950, an inhibitor that hindered NLRP3 inflammasome activation. It was found that the use of MCC950 also resulted in a major decrease in IL‐1β release, but a small amount of IL‐1β was still detected (Figure 6B). This suggests that IL‐1β in OSCC cells is mainly dependent on NLRP3 inflammasome, but there are other pathways involved in the process of IL‐1β maturation and secretion. The inflammatory environment may promote the proliferation and lymphatic vessel formation [32, 33]. To verify the direct role of IL‐1β on HLECs, we added human recombinant IL‐1β directly to the shSOAT1‐ supernatant of OSCC cells and used that for HLECs culturing and found that the tube formation ability of HLECs was significantly enhanced with increasing concentrations of IL‐1β (Figure 6C). When OSCC cells were pretreated with MCC950 and then cultured with HLECs, the ability of OSCC cells to promote tube formation was attenuated, repeatedly demonstrating the role of NLRP3 inflammasome activation in promoting tube formation in HLECs (Figure 6D). IL‐1R is the receptor for IL‐1β, of which IL‐1R‐1 has an important function [34]. To test the direct role of IL‐1β, we pretreated HLECs with IL‐1R‐1 neutralizing antibody (Figure 6E). When the receptors of HLECs were neutralized, subsequent stimulation with IL‐1β failed to promote the tube formation ability of HLECs as it did before, further illustrating that IL‐1β secreted from OSCC cells exerts a direct regulatory effect on lymphangiogenesis of HLECs through the IL‐1R‐1 receptor (Figure 6F).

## 4 Discussion

SOAT1 has become a promising targets for cancer therapy [7]. Recent studies have found that SOAT1 was upregulated in many cancers [9, 11, 35, 36, 37]. In our study, we also found higher expressions of SOAT1 in OSCC tissues as well as OSCC cells. The abnormal upregulation of SOAT1 enhanced the proliferation, migration, and invasion of Cal‐27 and HSC‐3 OSCC cells. Inhibiting SOAT1 expression could effectively hinder these malignant behaviors, highlighting its potential therapeutic role in the treatment of OSCC.

LN metastasis is the leading cause accounted for poor prognosis of OSCC patients [38]. The identification of novel as well as therapeutic targets aiming for lowing LN metastasis rate of OSCC is urgently needed. Lipid metabolism was proven involved in OSCC metastasis [39, 40]. In one study, the expression of 276 lipid metabolism‐related genes, functionally related to lipid uptake, triacylglycerols, phospholipids and sterols metabolism, was significantly upregulated in metastatic OSCC [41]. Fatty acid oxidation (FAO) was also detected involved in the invasion, lymphoinvasion, and epithelial‐mesenchymal transition (EMT) in OSCC cells. Higer FAO rates was also correlated to higher LN metastasis of OSCC [42]. The sphingolipid metabolic pathway was significantly activated in OSCC, and the role of key enzyme SPHK1 regulating OSCC tumor progression and promoting metastasis has also been elucidated [43]. In our study, SOAT1, a cholesterol esterification enzyme, has been proven positively related to LN metastasis as well. It showed a lower rate of LN metastasis in patients with lower SOAT1 expressions. What's more, less number and volume of lymph nodes was detected in shSOAT1‐ mice compared to that in shNC‐ mice, indicating that targeting SOAT1 is a promising therapeutic strategy to prevent OSCC from LN metastasis. Additional research evidence also supports the regulatory role of SOAT1 in tumor metastasis. Fu et al. reported that SOAT1 mediated EMT in hepatocellular carcinoma (HCC), and targeting SOAT1 via nootkatone could inhibit EMT of HCC in vitro and in vivo [37]. High intratumor SOAT1 expression also positively correlated to lymph node metastasis and indicated poor patient disease‐free survival and overall survival in colorectal cancer [36]. In a gallbladder cancer research, the expression of SOAT1 was found statistically significant with LN metastasis and TNM stages [35]. Lymphangiogenesis is considered as an important way for tumor progression and promoting its LN metastasis [44, 45]. In our study, we observed a higher density of lymphatic vessels in tumor tissues with higher SOAT1 expression, showing the positive correlation between SOAT1 and lymphangiogenesis. The evidence was firstly reported in gastric cancer. Zhu et al. found that a stronger expression of SOAT1 was detected in LN positive tissues than that in LN negative tissues [9], which also indicated the close relations of SOAT1 in regulating cancer‐associated lymphangiogenesis.

NLRP3, a member of inflammasome, has been noticed a tumor promotion role [12, 46, 47]. In OSCC, the role of NLRP3 was also elucidated. Upregulation of purinergic receptor P2X7 and NLRP3 inflammasome components were found in head and neck squamous carcinoma tissue as well as cells. Activation of P2X7 could increase the expression of NLRP3 and no harmful effects on cancer cell viability were noticed [48]. Huang et al. reported another potential role of NLRP3 in HNSCC. The positive correlation between NLRP3 inflammasome and cancer stem cells (CSCs) markers revealed that NLRP3 was associated with the carcinogenesis and CSCs self‐renewal activation [49]. NLRP3 inflammasome could be activated by many stimuli [31]. NLRP3 activation was reported in patients who received 5‐Fluorouracil (5‐FU), which led to 5‐FU chemoresistance of OSCC. Intracellular reactive oxygen species (ROS) levels may be the trigger to activate this protective NLRP3 upregulation for OSCC cells. Silencing of NLRP3 expression significantly inhibited OSCC cell proliferation and enhanced 5‐FU‐induced apoptosis of OSCC cells, indicating the potential role of NLRP3 in chemoresistance in OSCC [50]. In our study, we found that NLRP3 was relatively decreased when SOAT1 was knocked down in OSCC cells. When we reactivated the NLRP3 inflammasome in OSCC cells, the malignancy inhibited by SOAT1 knockdown was recovered, confirming that NLRP3 inflammasome as the downstream of SOAT1 play a vicious role for cancer progression.

Activation of NLRP3 inflammasome helps the maturation and release of IL‐1β and IL‐18, which were two classic inflammation cytokines. Chronic inflammation is an important event in carcinogenesis and tumor progression, and cancer‐related inflammation has been identified as the seventh hallmark of cancer [24, 51]. Inflammation environment in cancer has been proven promoting many cancerous aspects, including cancer stemness [49], proliferation [11], metastasis [36, 37], and tumor immune environment [52]. The chronic inflammation evolved in OSCC development has also been reported [53]. While whether OSCC cells promote the chronic inflammation was seldom known. In our study, more IL‐1β was released from OSCC cells, the release could be inhibited by SOAT1 knockdown. To further verify this process was mediated by NLRP3 inflammasome activation, MCC950 was applied to shNC‐ OSCC cells. The results showed a significant decrease of IL‐1β release, mechanically confirming that NLRP3‐dependent IL‐1β release was regulated by SOAT1. However, how SOAT1 impacted NLRP3 inflammasome activation was not discussed in our study. Lipid metabolism was closely related to NLRP3 inflammasome. In diabetic nephropathy, NLRP3 inflammasome activation could cause lipid accumulation in podocytes [54]. Free fatty acids (FFAs) produced could activate the TLR4 pathway, leading to TNF‐αsecretion by adipocytes. TNF‐α activates tumor necrosis factor receptor (TNFR) on recruited macrophages which, in combination with the TLR4 pathway, triggers NF‐κB nuclear import and production of NLRP3, pro‐IL‐1β and pro‐IL‐18, and then inflammasome activation and pro‐inflammatory cytokines release [55]. In cancer, tumor‐derived exosomal TRIM59 could convert macrophages to a pro‐tumor phenotype via regulating ABHD5 proteasomal degradation, and led to NLRP3 inflammasome activation to promote lung cancer progression [14]. Therefore, we speculate that SOAT1 may alter lipid metabolism within OSCC cells, which further promotes NLRP3 inflammasome activation and IL‐1β release. While the exact mechanisms still need to explore.

IL‐1β has been proven a notorious proinflammation cytokine in cancers. It involves in many aspects of cancer development, including cancer growth [26], stemness [56], EMT [57], angiogenesis [58], and metastasis [59]. Many studies have demonstrated its role in stimulating cancer‐related angiogenesis. Enhanced angiogenesis patterns, as evidenced by high vessel density in tumors and increased secretion of VEGF by the malignant cells, were observed in tumors secreting IL‐1β [60, 61]. Tumor‐related angiogenesis is inhibited if either IL‐1β or VEGF are neutralized, as IL‐1β and VEGF can work as an auto‐induction circuit and interact with bone marrow–derived VEGFR1^+^/IL‐1R1^+^ immature myeloid cells (MDSCs) and tissue‐resident endothelial cells [61]. While the regulation of IL‐1β in cancer‐related lymphangiogenesis was seldom reported. Since LN metastasis is main pathway for OSCC, we explore the role of IL‐1β in lymphangiogenesis. In our findings, IL‐1β could directly interact with LECs, and promote their tube formation capacity. When we neutralize IL‐1R‐1 in LECs, the promotion effect of IL‐1β was diminished, suggesting the direct role of IL‐1β in OSCC‐related lymphangiogenesis. While whether IL‐1β could regulate lymphangiogenesis via other pathways still need to discuss.

## 5 Conclusion

**Figure 7:**
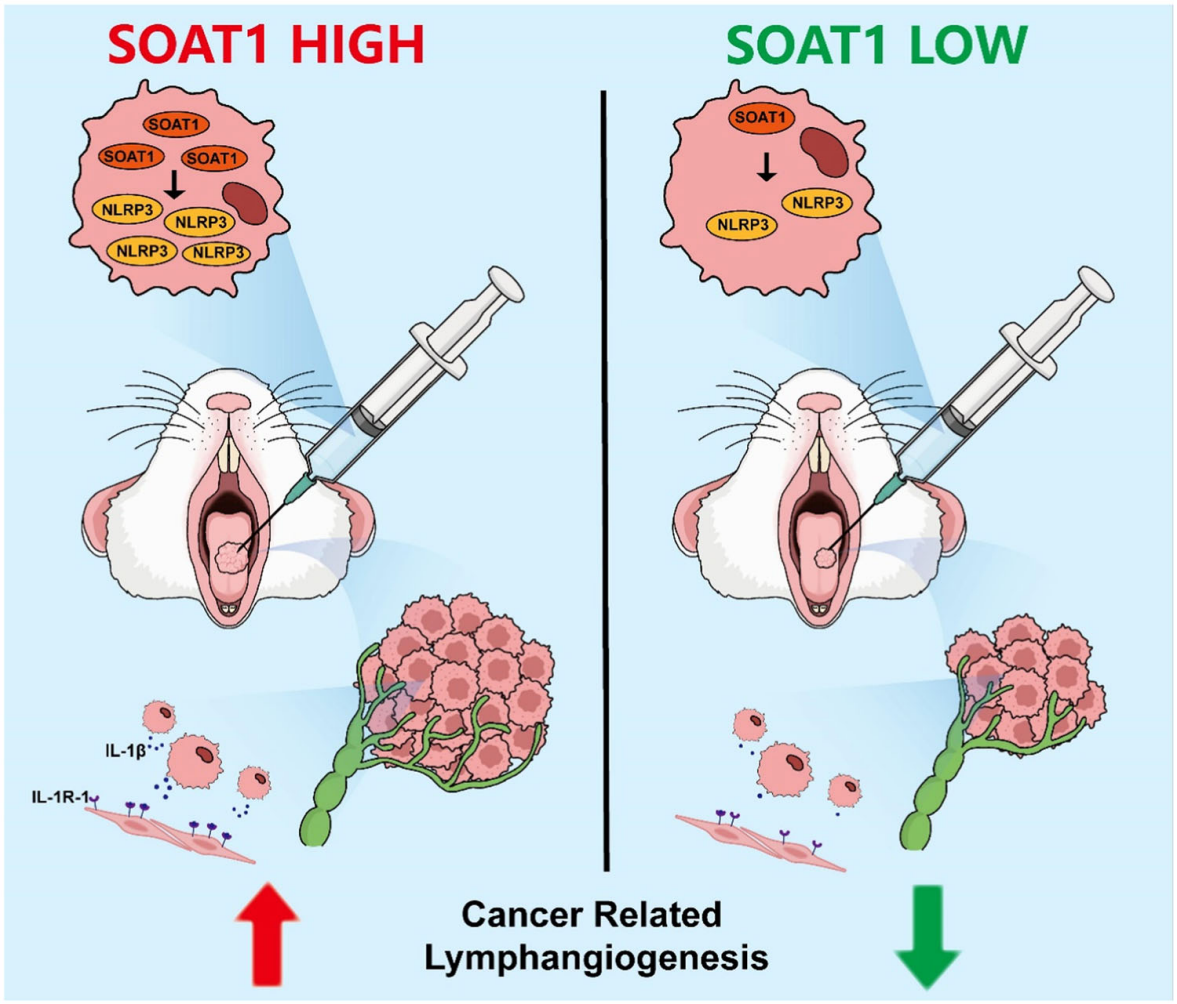
Schematic illustration showing the suggested mechanism that SOAT1 could activate NLRP3 inflammasome and promote OSCC cells to secret IL‐1β. IL‐1β could directly bind to IL‐1R‐1 expressed on HLECs and enhance cancer‐related lymphangiogenesis.

In conclusion, our study found out that SOAT1 expressions in OSCC tissues and cells were aberrantly upregulated. The higher expression of SOAT1 could promote OSCC cells on proliferation, migration, invasion in vitro. In vivo experiments demonstrated that SOAT1 could help tumor growth and promote OSCC metastasis to cervical lymph nodes. NLRP3 is the downstream of SOAT1, which is also associated with OSCC malignancy. Reactivation of NLRP3 inflammasome could recover impaired cancer cell migration and invasion abilities caused by SOAT1 knockdown. Besides, SOAT1‐mediated NLRP3 inflammasome activation in OSCC led to IL‐1β maturation and secretion. IL‐1β could directly bind to IL‐1R‐1 expressed in HLECs and promoted the tube formation capacities, which suggests a novel mechanism of cancer‐related lymphangiogenesis in OSCC (Figure 7).

## Author Contributions

Chengzhi Zhao, Jian Pan, and Dan Fan designed the experiment. Chengzhi Zhao and Yuhao Wang performed the experiments. Chengzhi Zhao, Zhishen Jiang, and Liru Hu analyzed the experimental data. Chengzhi Zhao wrote the manuscript, Chengzhi Zhao, Jian Pan, and Dan Fan revised the manuscript. All authors read and approved the final version of the manuscript.

## Conflicts of Interest

The authors declare no conflicts of interest.

## Supporting information


**Figure S1. The validation of siRNA knockdown efficiency. (A&B)** qRT‐PCR results showed SOAT1‐siRNA knockdown efficiency in Cal‐27 and HSC‐3 OSCC cells, respectively. **(C&D)** Western blot results showed SOAT1‐siRNA knockdown efficiency in Cal‐27 and HSC‐3 OSCC cells, respectively.

**Figure S2. The validation of shRNA transfection efficiency and knockdown efficiency. (A)** GFP fluorescence showed the efficiency of shRNA transfection into Cal‐27 and HSC‐3 cells. **(B&C)** qRT‐PCR results showed SOAT1‐shRNA knockdown efficiency in Cal‐27 and HSC‐3 cells, respectively. **(D&E)** Western blot results showed SOAT1‐shRNA knockdown efficiency in Cal‐27 and HSC‐3 cells, respectively.

**Figure S3. Gene expression and pathway enrichment changes in SOAT1‐knockdown Cal‐27 cells. (A)** PCA main component analysis in control and SOAT1‐knockdown Cal‐27 cells. **(B)** Heatmap of the differentiated genes of Cal‐27 cells after SOAT1 knockdown compared to control group. **(C&D)** GO and KEGG enrichment revealed the pathway alteration in Cal‐27 cells after SOAT1 knockdown compared to control group. **(E&F)** The gene expression heat map of the labelled genes in Toll‐like receptor and Nod‐like receptor pathway in control and SOAT1‐kncokdown Cal‐27 cells.

**Figure S4. SOAT promotes cervical LN metastasis in nude mice.** GFP fluorescence signals in the right neck regions in two mice groups via in vivo fluorescence image capture.

Supporting information.

## Data Availability

The authors have nothing to report.
