# Supplementary figures and images for: SOAT1 Activates NLRP3 Inflammasome to Promote Cancer‐Related Lymphangiogenesis and Metastasis via IL‐1β/IL‐1R‐1 Axis in Oral Squamous Cell Carcinoma
Source: Mol Carcinog. 2025 Mar 26;64(6):1039–56. doi: 10.1002/mc.23907 (PMC12074567; doi:10.1002/mc.23907)

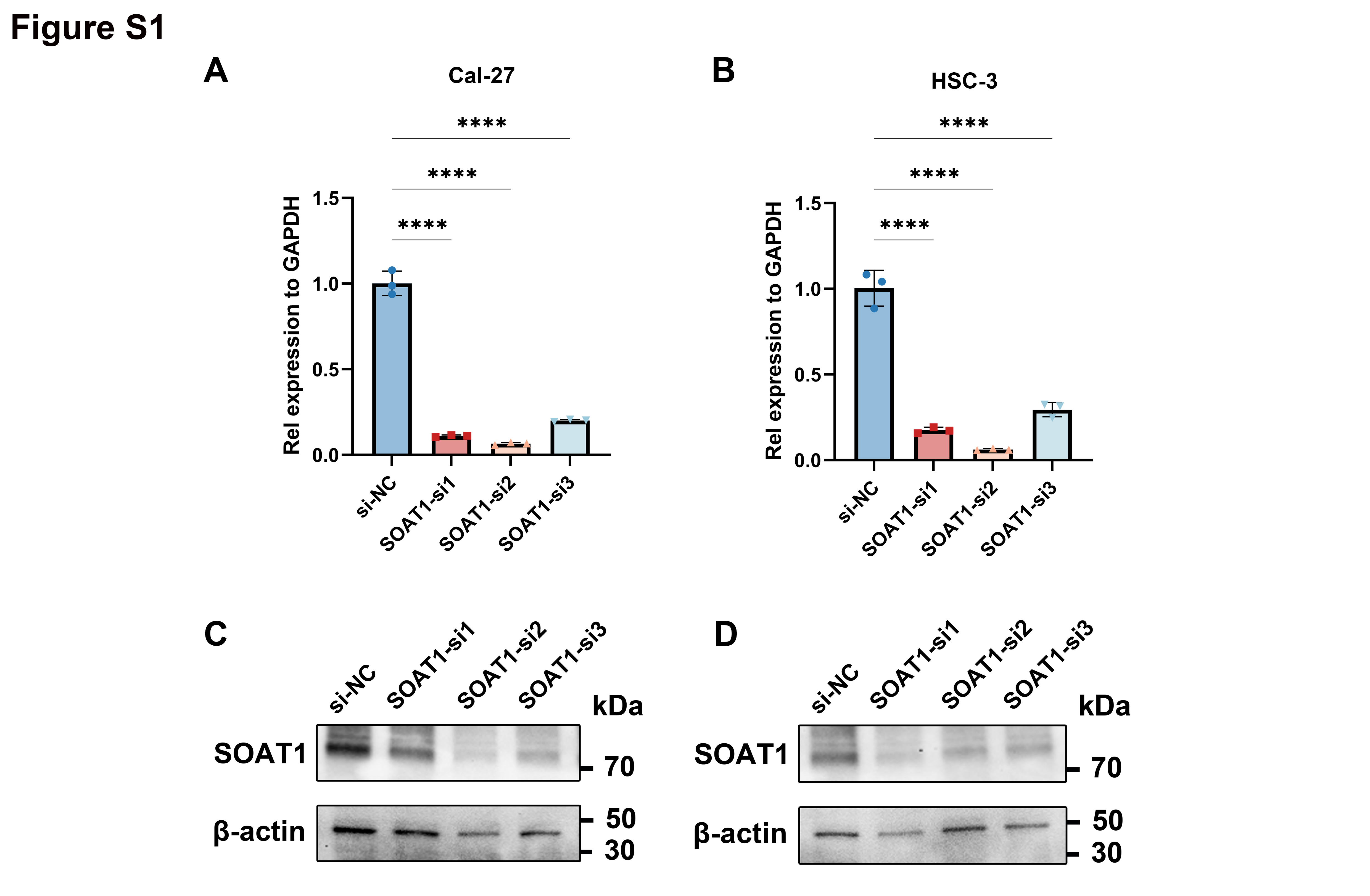

Supplement: Supplementary file 1 — Figure S1. The validation of siRNA knockdown efficiency. (A&B) qRT‐PCR results showed SOAT1‐siRNA knockdown efficiency in Cal‐27 and HSC‐3 OSCC cells, respectively. (C&D) Western blot results showed SOAT1‐siRNA knockdown efficiency in Cal‐27 and HSC‐3 OSCC cells, respectively. [file MC-64-1039-s005.jpg]

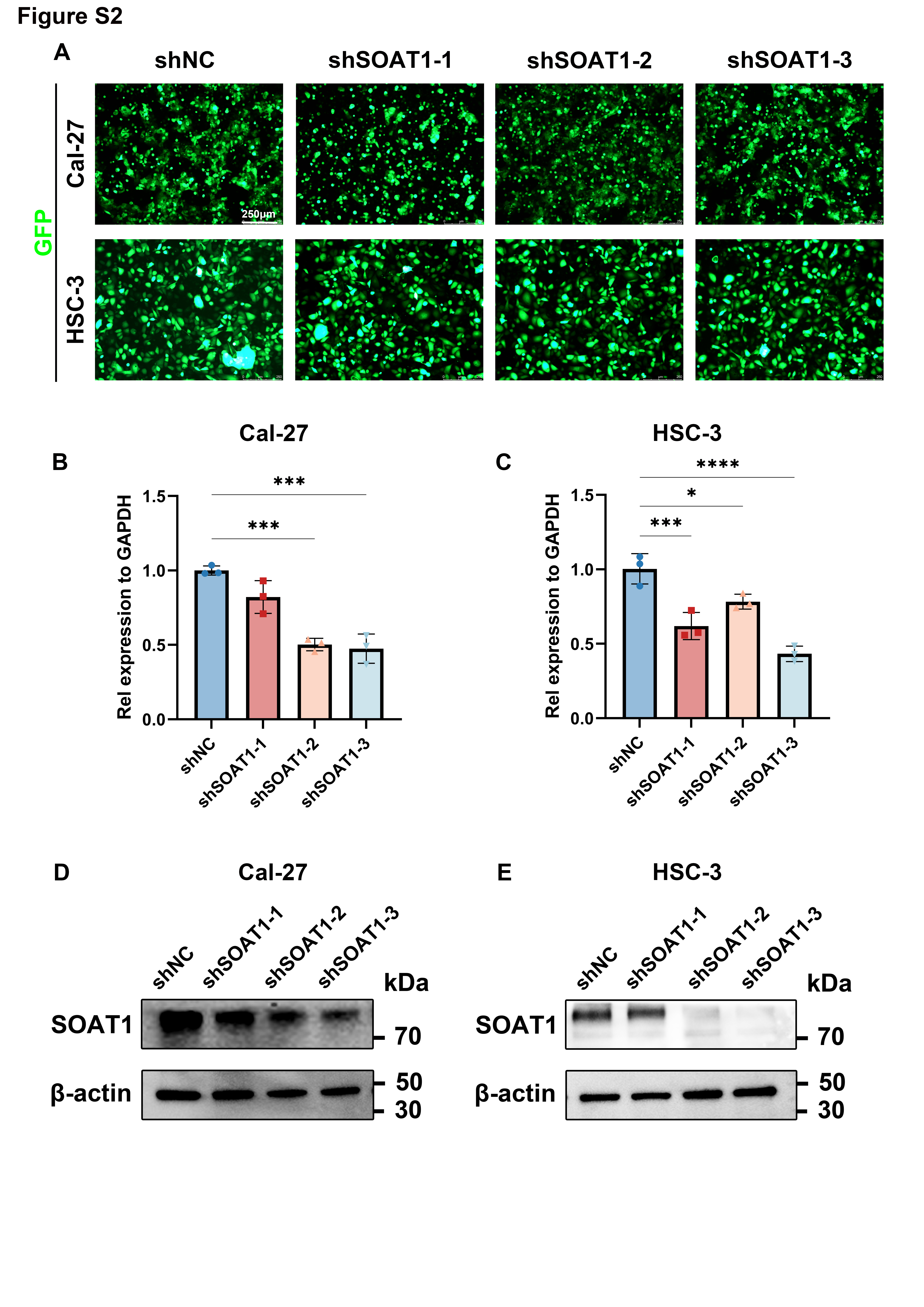

Supplement: Supplementary file 2 — Figure S2. The validation of shRNA transfection efficiency and knockdown efficiency. (A) GFP fluorescence showed the efficiency of shRNA transfection into Cal‐27 and HSC‐3 cells. (B&C) qRT‐PCR results showed SOAT1‐shRNA knockdown efficiency in Cal‐27 and HSC‐3 cells, respectively. (D&E) Western blot results showed SOAT1‐shRNA knockdown efficiency in Cal‐27 and HSC‐3 cells, respectively. [file MC-64-1039-s004.jpg]

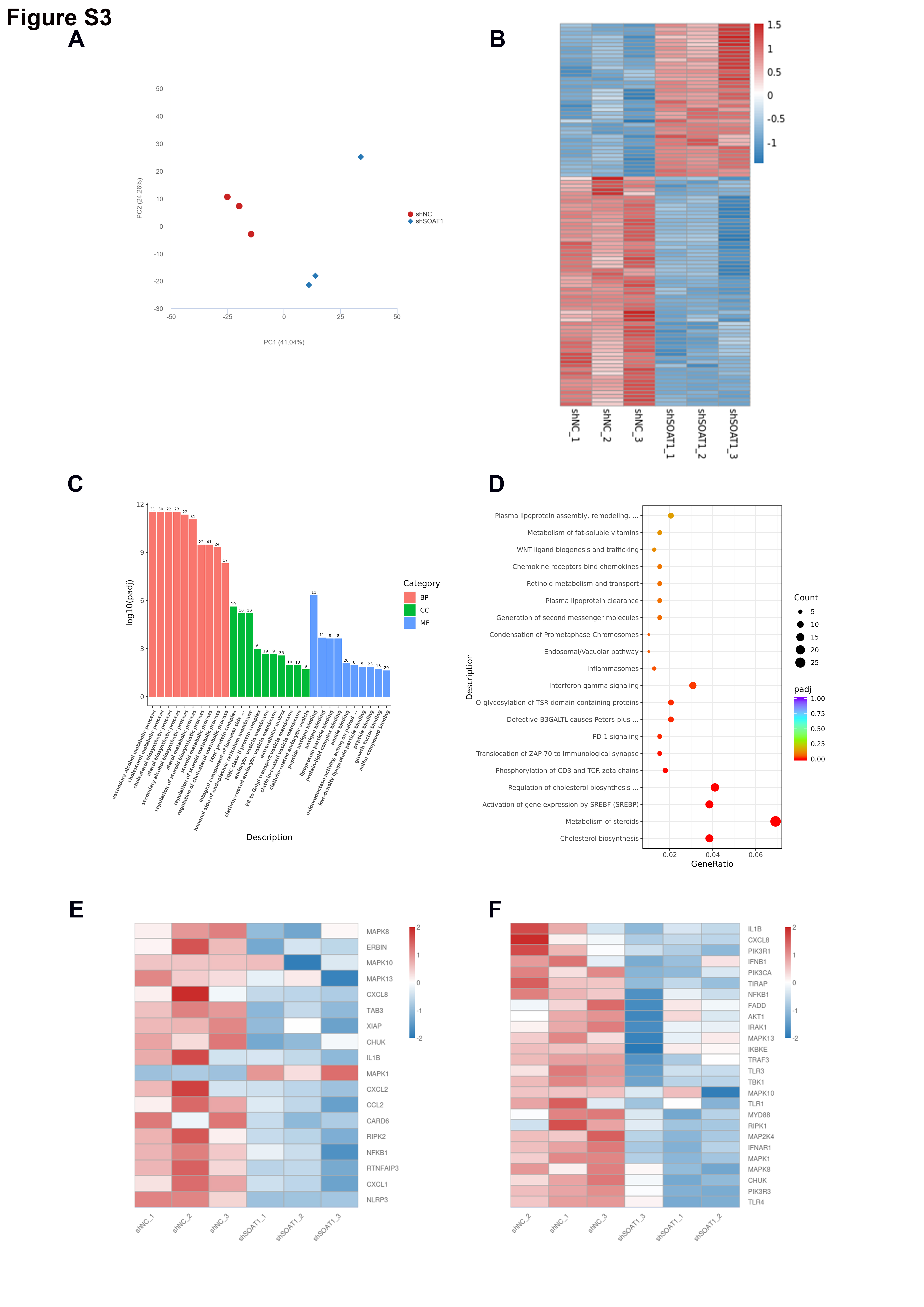

Supplement: Supplementary file 3 — Figure S3. Gene expression and pathway enrichment changes in SOAT1‐knockdown Cal‐27 cells. (A) PCA main component analysis in control and SOAT1‐knockdown Cal‐27 cells. (B) Heatmap of the differentiated genes of Cal‐27 cells after SOAT1 knockdown compared to control group. (C&D) GO and KEGG enrichment revealed the pathway alteration in Cal‐27 cells after SOAT1 knockdown compared to control group. (E&F) The gene expression heat map of the labelled genes in Toll‐like receptor and Nod‐like receptor pathway in control and SOAT1‐kncokdown Cal‐27 cells. [file MC-64-1039-s003.jpg]

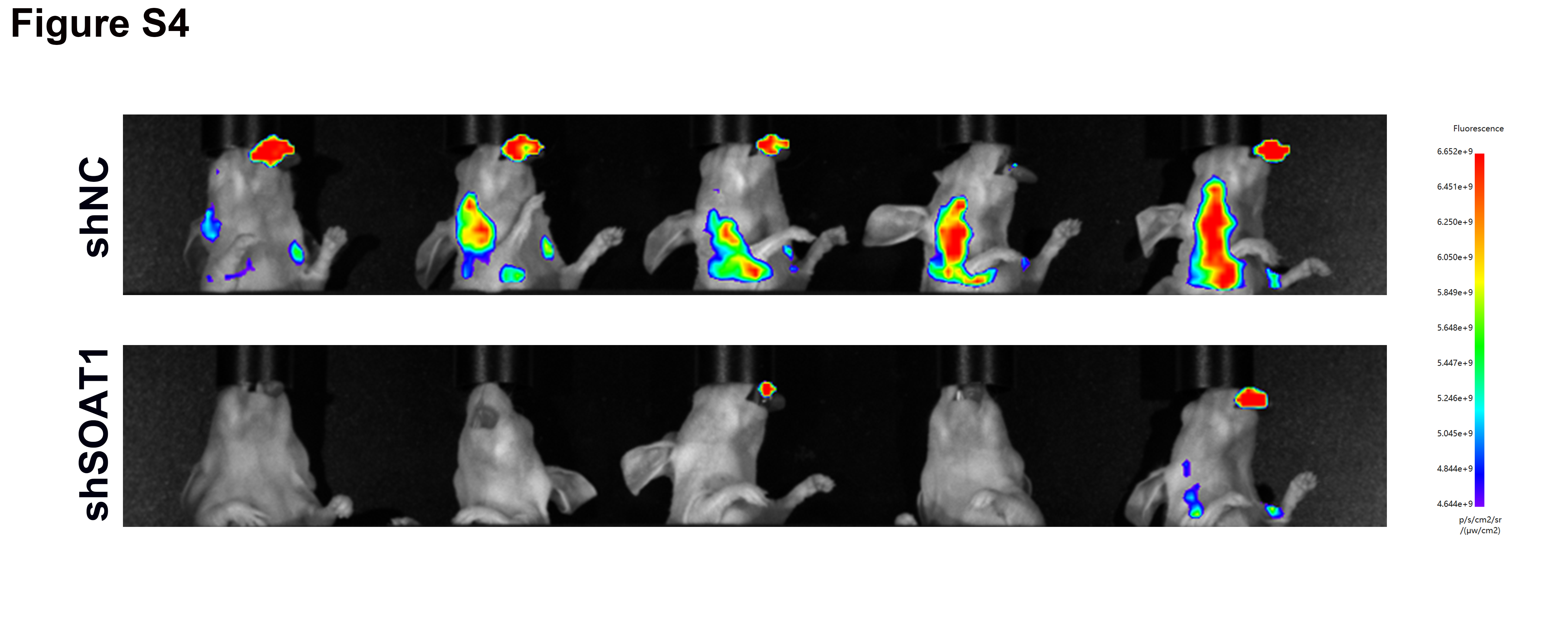

Supplement: Supplementary file 4 — Figure S4. SOAT promotes cervical LN metastasis in nude mice. GFP fluorescence signals in the right neck regions in two mice groups via in vivo fluorescence image capture. [file MC-64-1039-s002.jpg]
