# Supplementary material for: SOAT1 Activates NLRP3 Inflammasome to Promote Cancer‐Related Lymphangiogenesis and Metastasis via IL‐1β/IL‐1R‐1 Axis in Oral Squamous Cell Carcinoma
Source: Mol Carcinog. 2025 Mar 26;64(6):1039–56. doi: 10.1002/mc.23907 (PMC12074567; doi:10.1002/mc.23907)
Supplement: Supplementary file 5 — Supporting information. [file MC-64-1039-s001.docx]

**Supporting Information**

Figure S1

The validation of siRNA knockdown efficiency. (A&B) qRT-PCR results showed three siRNA sequence could effectively knockdown the mRNA expression of SOAT1 in Cal-27 and HSC-3 OSCC cells, respectively. (C&D) Western blot results showed three siRNA sequence could effectively knockdown the mRNA expression of SOAT1 in Cal-27 and HSC-3 OSCC cells, respectively.


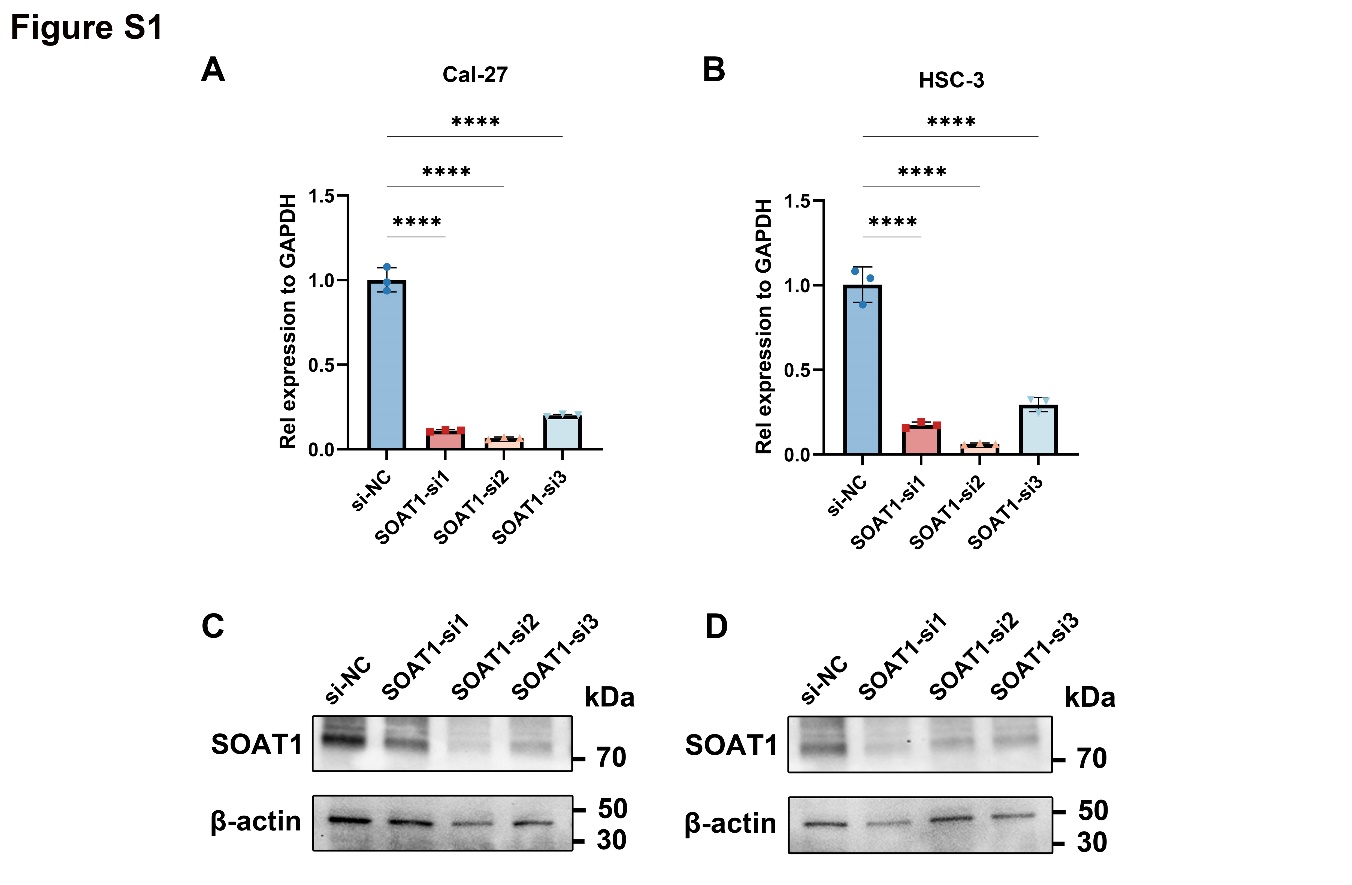


Figure S2

The validation of shRNA transfection efficiency and knockdown efficiency. (A) GFP fluorescence showed that all shRNA were successfully transfected into Cal-27 and HSC-3 OSCC cells. (B&C) qRT-PCR results showed shRNA sequence could effectively knockdown the mRNA expression of SOAT1 in Cal-27 and HSC-3 OSCC cells, respectively. (D&E) Western blot results showed shRNA sequence could effectively knockdown the mRNA expression of SOAT1 in Cal-27 and HSC-3 OSCC cells, respectively.


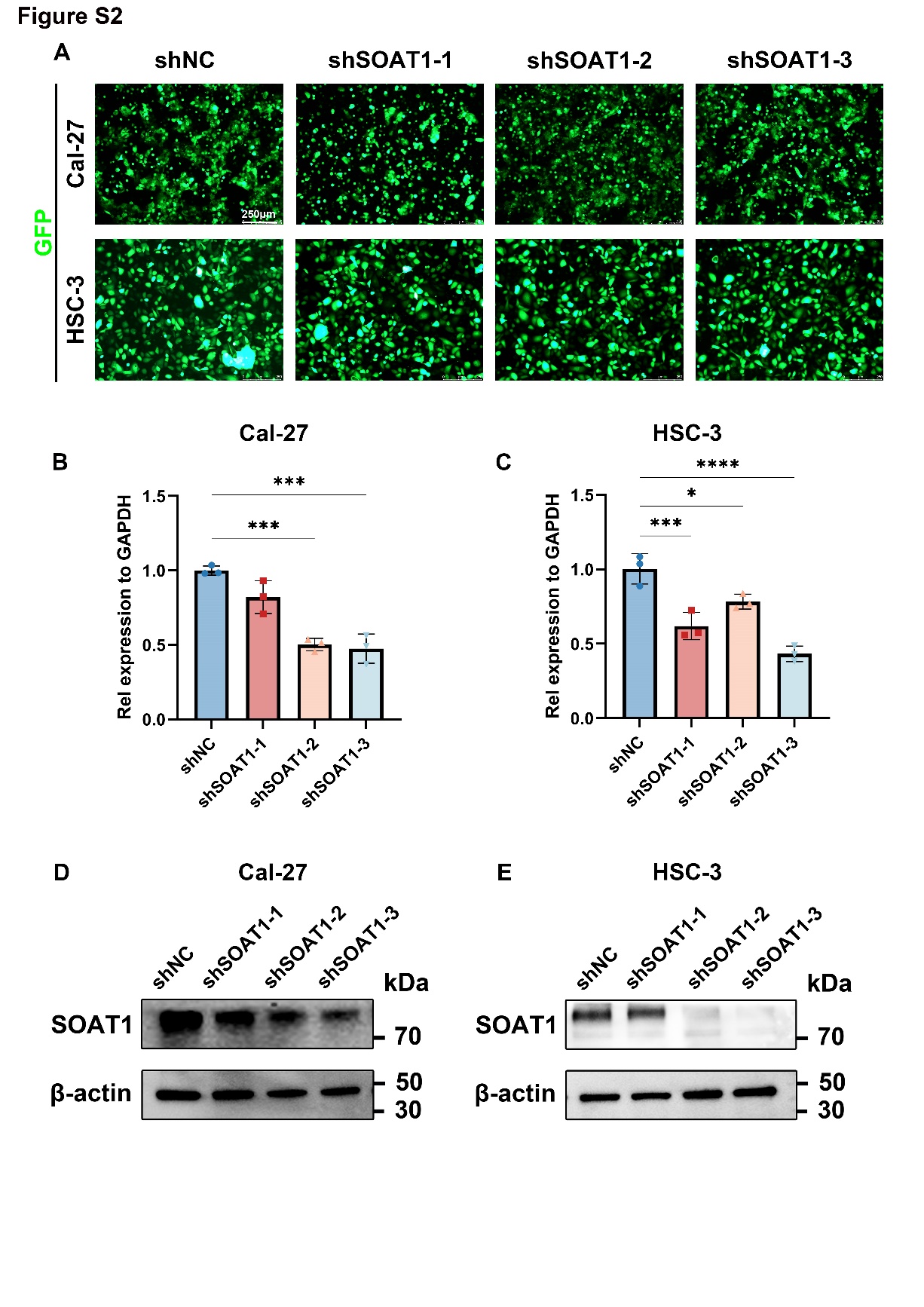


Figure S3

Gene expression and pathway enrichment changes in SOAT1-knockdown Cal-27 OSCC cells. (A) PCA main component analysis revealed SOAT1 knockdown made a difference on gene expression in Cal-27 OSCC cells. (B) Heatmap of the differentiated genes (DEGs) following SOAT1 knockdown in Cal-27 OSCC cells. (C&D) GO and KEGG enrichment revealed that SOAT1 knockdown led to lipid metabolism disturbance in Cal-27 OSCC cells. (E&F) The expression levels in shNC- and shSOAT1- Cal-27 OSCC cells of the labelled genes in Toll-like receptor pathway and Nod-like receptor pathway.


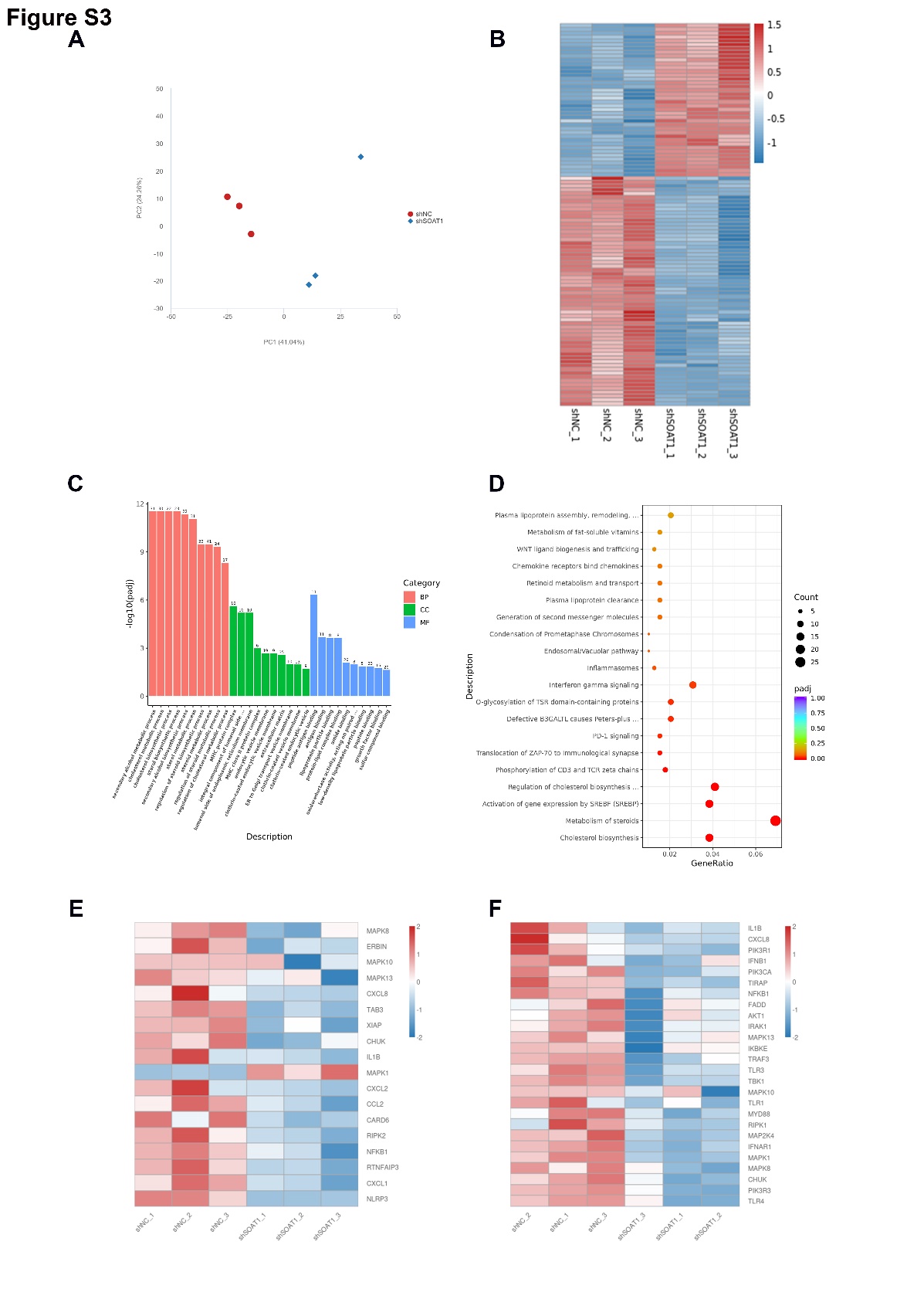


Figure S4

SOAT promotes cervical LN metastasis in nude mice. shNC- mice showed stronger GFP fluorescence signals in the right neck regions compared to that in shSOAT1- mice via in vivo fluorescence image capture.


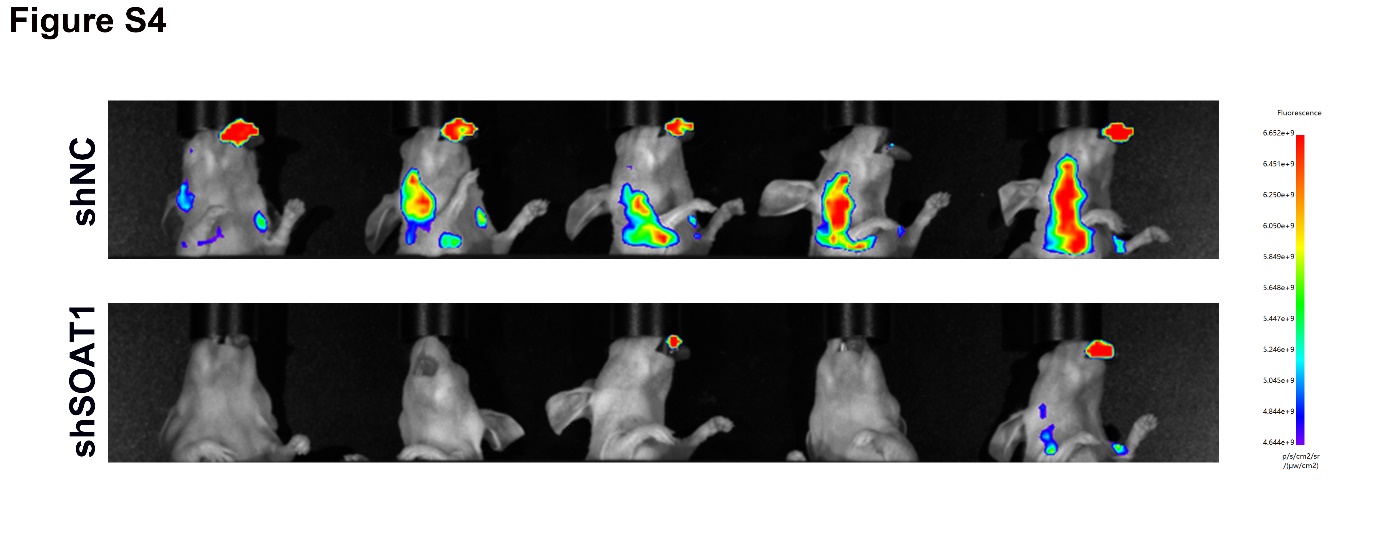


**Table S1**

Sequences of siRNA and shRNA.

| **Probes** | **Sequence (5’-3’)** |
| --- | --- |
| SOAT1-si1 | UAAUCUACUACAAGUGUGC |
| SOAT1-si2 | UAGUUGGAGUAUGACGUGG |
| SOAT1-si3 | AUCAGAACAUUCCAAAUCGGC |
| shSOAT1-1 | GCCGATTTGGAATGTTCTGAT |
| shSOAT1-2 | CCACGTCATACTCCAACTATT |
| shSOAT1-3 | GAACGTGCCTCGGGTACTAAA |

**Table S2**

Primers for qRT-PCR.

| **Gene** | **Sequence (5’-3’)** | **Sequence (3’-5’)** |
| --- | --- | --- |
| *GAPDH* | ACAACTTTGGTATCGTGGAAGG | GCCATCACGCCACAGTTTC |
| *SOAT1* | CCACTGGTCCAGATGAGTTTAG | GGGAACATGCAGAGTACCTTT |
